# Microbiome Profiling from Fecal Immunochemical Test Reveals Microbial Signatures with Potential for Colorectal Cancer Screening

**DOI:** 10.3390/cancers15010120

**Published:** 2022-12-25

**Authors:** Olfat Khannous-Lleiffe, Jesse R. Willis, Ester Saus, Victor Moreno, Sergi Castellví-Bel, Toni Gabaldón, on behalf of the CRIPREV Consortium

**Affiliations:** 1 Barcelona Supercomputing Center (BSC-CNS), Carrer de Jordi Girona, 29, 31, 08034 Barcelona, Spain; 2 Institute for Research in Biomedicine (IRB), Carrer de Baldiri Reixac, 10, 08028 Barcelona, Spain; 3 Catalan Institute of Oncology (ICO), L’Hospitalet de Llobregat, 08908 Barcelona, Spain; 4 Bellvitge Biomedical Research Institute (IDIBELL), L’Hospitalet de Llobregat, 08908 Barcelona, Spain; 5 Consortium for Biomedical Research in Epidemiology and Public Health (CIBERESP), Av. de Monforte de Lemos, 3–5, 28029 Madrid, Spain; 6 Gastroenterology Department, University of Barcelona, 08036 Barcelona, Spain; 7 Gastroenterology Department, Institut d’Investigacions Biomèdiques August Pi i Sunyer (IDIBAPS), Centro de Investigación Biomédica en Red de Enfermedades Hepáticas y Digestivas (CIBERehd), Hospital Clínic, 08036 Barcelona, Spain; 8 Institució Catalana de Recerca i Estudis Avançats (ICREA), Pg. Lluís Companys 23, 08010 Barcelona, Spain; 9 Centro Investigación Biomédica En Red de Enfermedades Infecciosas (CIBERINFEC), 08028 Barcelona, Spain

**Keywords:** colorectal cancer, microbiome, 16S rRNA sequencing, screening, diagnosis

## Abstract

**Simple Summary:**

Colorectal cancer (CRC) is a global healthcare challenge that involves both genetic and environmental factors. Several pieces of evidence suggest that alterations of the gut microbiome can influence CRC development. In the present study we analyzed 16S rRNA sequencing data from fecal immunochemical test (FIT) samples from a large cohort, observing a predictive potential of the microbiome, revealing changes along the path from healthy tissue to carcinoma. Our work has implications in the understanding of the roles of microbes on the adenoma to carcinoma progression and opens the door to an improvement of the current CRC screening programmes.

**Abstract:**

Colorectal cancer (CRC) is the third most common cancer and the second leading cause of cancer deaths worldwide. Early diagnosis of CRC, which saves lives and enables better outcomes, is generally implemented through a two-step population screening approach based on the use of Fecal Immunochemical Test (FIT) followed by colonoscopy if the test is positive. However, the FIT step has a high false positive rate, and there is a need for new predictive biomarkers to better prioritize cases for colonoscopy. Here we used 16S rRNA metabarcoding from FIT positive samples to uncover microbial taxa, taxon co-occurrence and metabolic features significantly associated with different colonoscopy outcomes, underscoring a predictive potential and revealing changes along the path from healthy tissue to carcinoma. Finally, we used machine learning to develop a two-phase classifier which reduces the current false positive rate while maximizing the inclusion of CRC and clinically relevant samples.

## 1. Introduction

Colorectal cancer (CRC) is the third most common cancer type and the second leading cause of cancer-related deaths worldwide [1], accounting for nearly 900,000 deaths each year. This malignant disease develops from the pathological transformation of normal colonic epithelium to adenomatous polyps, which ultimately leads to invasive cancer. This process is gradual and involves the accumulation of genetic and/or epigenetic alterations [2]. CRC incidence increases with economic development and Westernization of dietary and lifestyle habits, hinting at a significant effect of environmental and lifestyle factors, likely in combination with genetic predisposition [3]. In this regard, a growing body of evidence has linked alterations of the gastrointestinal tract microbiota with CRC development [4]. Earlier research has shown that alterations in the gut microbiota may influence colon tumorigenesis [5] through chronic inflammation or the production of carcinogenic compounds [6]. Differences in the relative abundances of some microbial species or genera have been found when comparing paired tumor and normal tissues, or fecal samples from CRC patients and healthy subjects [7,8]. 

Diagnosis of CRC is challenging and involves a complex process that usually starts with the detection of the first symptoms by the patient, and is followed by clinical diagnostic procedures, mainly based on colonoscopy. The implementation of preventive measures and early diagnosis of CRC can save many lives [9,10] and routine screening of asymptomatic populations following an age-selected criteria has been implemented in many countries. Current CRC screening in the vast majority of Western countries consists of a two-step procedure with a non-invasive test (most commonly a fecal immunochemical test (FIT) for quantification of occult hemoglobin in the stool) followed by colonoscopy if the test is positive (FIT-positive, or more accurately, above a given threshold of hemoglobin concentration) [11,12]. This approach is effective but results in a high rate of false positives (around 65% FIT-positive samples reveal no clinically relevant feature at colonoscopy) at the first step and many unnecessary colonoscopies, with a FIT sensitivity of around 35% [13]. Colonoscopy is an invasive, expensive and time-consuming procedure, and hence additional biomarkers that could better stratify individuals with higher risk for CRC or premalignant lesions to undergo a colonic examination would significantly reduce health-care costs. Much current research is directed towards finding additional criteria, such as risk factors and alternative biomarkers to be considered by the decision algorithms used to personalize positive FIT testing to colonoscopy. To search for potential predictive biomarkers present in FIT samples and to shed light on the potential roles of the gut microbiome in CRC development, we performed microbiome profiling using targeted sequencing of the 16S V3-V4 region from DNA extracted directly from FIT containers collected within the population-based organized screening program implemented in Catalonia, Spain [14]. We analyzed a total of 2889 FIT-positive samples and assessed their microbial composition and metabolic potential, and how they varied across samples with different colonoscopy results (i.e., different diagnostic outcome after colonoscopy exploration, including, among others, the absence of any clinical feature, the presence of lesions and their risk, the presence of colorectal cancer, and the presence of polyps). 

## 2. Materials and Methods

Our study followed the Strengthening the Organization and Reporting of Microbiome Studies (STORMS) checklist (Data S1).

### 2.1. Sample Collection and Subjects

A total of 2889 FIT-positive (>20 μg hemoglobin/g feces) samples recruited in two rounds (2009 and 2017–2019) from asymptomatic participants from the Catalan CRC screening program were analysed. Individuals were selected within the age criteria implemented by the screening programme (50 to 69 years old) and the diagnosis and sex selection were based on an ideal balanced dataset (aimed to obtain equal numbers within each class). Collected metadata comprised six different clinical variables for each sample, including the diagnosis after colonoscopy evaluation (Data S2), the number of polyps, the FIT value (μg of hemoglobin/g of feces), the hospital at which the sample was collected, and the donor’s sex and age. The considered colonoscopy diagnoses were negative (N), colorectal cancer (CRC) and different lesions that can be relevant in CRC development: carcinoma in situ (CIS), high risk lesion (HRL), intermediate risk lesion (IRL), low risk lesion (LRL) and lesion not associated to risk (LNAR) [15] (Table S1). Additionally, we classified the samples into two groups according to the clinical relevance of the colonoscopy-based diagnosis [16]: CRC, CIS, HRL and IRL were considered clinically relevant (CR) lesions (indeed, they are the goal of CRC screening programs), and N, LNAR and LRL as non-clinically relevant (non-CR) lesions (Table S1). Individuals with inflammatory bowel disease or polyposis were excluded from the study. Our study was approved by the institutional ethical committees of the involved institutions and informed consent was obtained from the participants. 

### 2.2. DNA Extraction and 16S Sequencing

Aliquots of 500 μL of buffer contained in FIT collection devices (OC-Sensor, Eiken Chemical Co., Tokyo, Japan) were prepared in a test tube and stored at −80 °C until further processing. DNA was extracted from FIT samples using the DNeasy PowerLyzer PowerSoil Kit (Qiagen, ref. QIA12855) following manufacturer’s instructions. The extraction tubes were agitated twice in a 96-well plate using the TissueLyser II (Qiagen) at 30 Hz/s for 5 min.

Four μL of each DNA sample were used to amplify the V3–V4 regions of the bacterial 16S ribosomal RNA gene, using the following universal primers in a limited cycle PCR: V3-V4-Forward (5′-TCGTCGGCAGCGTCAGATGTGTATAAGAGACAGCCTACGGGNGGCWGCAG-3′) and V3-V4-Reverse (5′-GTCTCGTGGGCTCGGAGATGTGTATAAGAGACAGGACTACHVGGGTATCTAATCC-3′). To prevent unbalanced base composition in further MiSeq sequencing, we shifted sequencing phases by adding a variable number of bases (from 0 to 3) as spacers to both forward and reverse primers (we used a total of 4 forward and 4 reverse primers). The PCR was performed in 10 μL volume reactions with 0.2 μM primer concentration and using the Kapa HiFi HotStart Ready Mix (Roche, ref. KK2602). Cycling conditions were initial denaturation of 3 min at 95 °C followed by 25 cycles of 95 °C for 30 s, 55 °C for 30 s, and 72 °C for 30 s, ending with a final elongation step of 5 min at 72 °C. 

After the first PCR step, water was added to a total volume of 50 μL and reactions were purified using AMPure XP beads (Beckman Coulter) with a 0.9X ratio according to manufacturer’s instructions. PCR products were eluted from the magnetic beads with 32 μL of Buffer EB (Qiagen) and 30 μL of the eluate were transferred to a fresh 96-well plate. The primers used in the first PCR contained overhangs allowing the addition of full-length Nextera adapters with barcodes for multiplex sequencing in a second PCR step, resulting in sequencing ready libraries. To do so, 5 μL of the first amplification was used as template for the second PCR with Nextera XT v2 adaptor primers in a final volume of 50 μL using the same PCR mix and thermal profile as for the first PCR but for only 8 cycles. After the second PCR, 25 μL of the final product was used for purification and normalization with the SequalPrep normalization kit (Invitrogen), according to the manufacturer’s protocol. Libraries were eluted in 20 μL and pooled for sequencing. 

Final pools were quantified by qPCR using the Kapa library quantification kit for Illumina Platforms (Kapa Biosystems) on an ABI 7900HT real-time cycler (Applied Biosystems). Sequencing was performed in the Illumina MiSeq with 2 × 300 bp reads using v3 chemistry with a loading concentration of 18 pM. To increase the diversity of the sequences, 10% of PhIX control libraries were spiked in. 

Two bacterial mock communities were obtained from the BEI Resources of the Human Microbiome Project (HM-276D and HM-277D), each containing genomic DNA of ribosomal operons from 20 bacterial species [17]. Mock DNAs were amplified and sequenced in the same manner as all other FIT samples. Negative controls of the DNA extraction and PCR amplification steps were also included in parallel, using the same conditions and reagents. These negative controls provided no visible band or quantifiable DNA amounts by Bioanalyzer, whereas all our samples provided clearly visible bands after 25 cycles. 

### 2.3. Microbiome Analysis

We used the dada2 (v. 1.10.1) pipeline [18] to obtain an amplicon sequence variants (ASV) table for each of the sequencing runs separately. The quality profiles of forward and reverse sequencing reads were examined using the plotQualityProfile function of dada2 and, according to these plots, low-quality sequencing reads were filtered and trimmed using the filterAndTrim function. We obtained a matrix with learned error rates with the learnErrors dada2 function. We performed dereplication (combining identical sequencing reads into unique sequences), sample inference (from the matrix of estimated learning error rates) and merged paired reads to obtain full denoised sequences. From these, chimeric sequences were removed. Taxonomy was assigned to ASVs by mapping to the SILVA 16s rRNA database (v. 132) [19]. Negative controls (non-template samples) and positive controls (mock microbial communities comprising a mixture of 20 strains with known proportions) were sequenced and analyzed in each of the runs to assess the possible contamination background and evaluate the accuracy of the pipeline. We obtained ASV and taxonomy tables for each run separately, and then merged the results. Samples without metadata information and the controls were discarded in further analyses. 

We reconstructed a phylogenetic tree by using the phangorn (v. 2.5.5) [20] and Decipher R packages (v 2.10.2) [21] and integrated it with the merged ASV and Taxonomy tables and their assigned metadata creating a phyloseq (v. 1.26.1) object [22]. We characterized alpha diversity metrics including Observed index, Shannon, Simpson, InvSimpson, PD Chao1, ACE and standard error measures such as se.Chao1 and se.ACE using the estimate_richness function of the phyloseq package. Using the picante package (v. 1.8.1) we computed Faith’s phylogenetic diversity, an alpha diversity metric that incorporates branch lengths of the phylogenetic tree. Additionally, we calculated different distance metrics based on the differences in taxonomic composition between samples using the Phyloseq and Vegan (v. 2.5–6) [23] packages. These metrics include Jensen-Shannon Divergence (JSD), Weighted-Unifrac, Unweighted-unifrac, Bray-Curtis dissimilarity, Jaccard and Canberra. We also computed Aitchison distances between samples using the cmultRepl and codaSeq.clr functions from the CodaSeq (v. 0.99.6) [24] and zCompositions (v. 1.3.4) [25] packages. Normalization was performed by transforming counts to centered log-ratios (clr) [26]. We performed multiplicative simple zero replacement as implemented in the cmultRepl function of the zCompositions package (v. 1.3.4) (indicating method = “CZM”). Samples with fewer than 1000 reads and taxa that appeared in fewer than 10 samples and at low abundances (fewer than 100 reads) were filtered out. Finally, we agglomerated taxa at each taxonomic rank to study trends at different taxonomic depths.

We made a comparison of our overall microbiome profiles with samples studied in a previous study [27]. We treated the samples from their 2 × 300 pb cycle run by applying the same procedure state in the present section. 

### 2.4. Statistical Analysis

We assessed associations between clinical variables and the overall microbial composition of the samples by performing permutational multivariate analysis of variance (PERMANOVA) using the adonis function from the Vegan R package (v. 2.5–6) with the seven distance metrics mentioned above. Diagnosis, sex and age variables were considered as covariates. Additionally, we performed an analysis of similarities (ANOSIM) test using the anosim function from the Vegan R package to assess differences between and within groups. 

We performed a differential abundance analysis using clr data for the different taxonomic ranks across various clinical variables using linear models implemented in the R package lme4 (v. 1.1–21) [28]. We built a linear model including diagnosis (Dx), hospital, sex, age, number of polyps and FIT value as fixed effects, and the sequencing run as a random effect to account for possible batch effects: tax_element~Dx + hospital + sex + age + number_polyps + FIT_value + (1|run). This linear model was evaluated considering all the diagnoses, but also made a comparison of CRC versus non-CRC samples by changing all other diagnoses to “others”. A second linear model was applied that considered as fixed effect a variable called risk instead of the diagnosis in order to assess the differences between samples with CR or non-CR colonoscopy, as defined above (Table S1). 

We applied analysis of variance (ANOVA) to assess the significance for each of the fixed effects included in the models using the Car R package (v. 3.0–6) [29]. To assess differences between groups, we performed multiple comparisons to the results obtained in the linear models using the Tukey test in the function glht from the multcomp R package (v. 1.4–12) [30]. We applied Bonferroni as a multiple testing correction as implemented in the summary.glht function of the multcomp package, and statistical significance was defined at *p* values lower than 0.05. In addition, we used the selbal package (v. 0.1.0) [31] to study groups of taxa (balances) with potential predictive power for CRC status. 

### 2.5. Co-Occurrence and Networks

Co-occurrence networks for microbial species were inferred and represented for each of the diagnostic groups, considering the top 50 taxa and using the SpiecEasi R package (v. 1.1.0) [32]. We used neighborhood selection based on penalized regression as the graphical model inference. The resulting networks, following the path transition from healthy colon (N) to cancer (CRC), were compared by computing hamming distances with the netdist function from the R package nettools (v. 1.1.0). We represented the weights of the correlations of the co-occurrence networks by using the chordDiagram function from the circlize package (v. 0.4.12). 

We also calculated taxa correlation matrices for each diagnosis group by using the function corr.test from the R psych package (v. 2.0.12) and using the Spearman method, adjusting for multiple comparisons with the Holm-Bonferroni method. The significance threshold was set at p.adjust < 0.05. 

### 2.6. Genome Content Inference

Given the ASV and taxonomy tables in the phyloseq object, we applied the t4f function from the themetagenomics package (v. 1.0.0) [33] to predict the functional content in terms of functional genes (kegg orthologous groups (OGs), which are families of genes that descent from a common ancestral gene and that generally perform similar functions). Then, we applied a linear model (ortholog~Dx + hospital + sex + age + number_polyps + FIT_value + (1|run)) to determine OGs that were significantly differentially abundant according to the diagnosis, and a multiple comparison test (Tukey) correcting by Bonferroni. From these differentially abundant OGs, we extracted all the functional pathways in which they were involved and performed a test for pathway enrichment only considering pathways with 10 or more predicted OGs and having at least 10% of their OGs being differentially abundant. Using custom scripts and text mining tools implemented in the easyPubMed R package (v.2.13) [34], we retrieved pubmed articles in which these pathways appeared related to CRC.

### 2.7. Machine Learning Classification

We developed a predictive model based on a two-phase classification using a neural network (NN) algorithm implemented in the caret package (v. 6.0–85) [35]. For each phase we trained a random 75% of the data with a 10-fold cross validation and tested with the remaining samples. The process was repeated 100 times to avoid “lucky” splits and to evaluate the variability in predictive performance. We performed a feature selection based on the differential abundance results including taxa found as having significantly different abundances in our study and incorporating FIT-value, age and sex variables. Samples with missing values for the considered metadata were removed. Taxa abundances were included as clr. The two-phase classifier proceeds as follows: in the first phase the method classifies CRC vs. non-CRC samples. Samples that are classified as non-CRC in the first phase are subjected to a second model that classifies CR vs. non-CR samples. At the end of the two-phase classification, the mean percentage of misclassified CRC and CR samples was calculated, and the performance of the model was evaluated. 

To validate our strategy we built a model training with all the CRIPREV samples and tested it in two independent datasets: a cohort from the USA [36,37] and 100 extra samples from the same Catalan screening. For the USA cohort, we applied the Catalan hemoglobin threshold (>20 μg of hemoglobin/g of feces) to select the FIT-positive samples to include in the validation. We processed their raw data following the same methodology as in our study (see Microbiome analysis, Materials and Methods). We unfortunately could not assign *Bacteroides fragilis*, likely because that study only used the V4 region of the 16S rRNA gene as compared to V3-V4 in our study. 

We assessed possible subsets of taxa with classification potential by using the 100 extra samples from the same local screening. We identified a total of 27 taxa, found as differentially abundant in both the CRC vs. others and CR vs. non-CR comparisons, intersecting between the CRIPREV project and these extra samples, that are those included in the results presented here. We assessed different combinations of the taxa, considering the effect size observed in our statistical test. We defined top and down taxa from the list, per each phase, and made an assessment of subsets of taxa as follows: 4 taxa from the top of the list (50 random combinations), 4 taxa from the bottom of the list (50 random combinations), 4 random taxa (50 random combinations), 2 taxa from the top of the list (all the possible combinations), 2 taxa from the bottom of the list (all the possible combinations), 1 taxa from the top of the list (all the possible combinations) and 1 taxa from the bottom of the list (all the possible combinations). 

We tested a total of 948 models using our validation set. We filtered the models based on some classification metrics: AUC1 >= 0.55, specificity1 > 0.2, AUC2 > 0.5 and specificity2 > 0. 

ROC curves were represented using the package pROC (v 1.16.1) [38]. 

## 3. Results

### 3.1. 16S Metabarcoding from FIT Samples Is a Valid Proxy for Gut Microbiome

To assess the diagnostic and research potential of microbiome analyses performed on FIT samples collected within currently ongoing CRC screening programs, we enrolled asymptomatic participants of the Catalan CRC screening program that had a FIT-positive test. We froze their FIT cartridges until the results from the colonoscopy examination were obtained. These outcomes were categorized into clinically relevant (CR) lesions -including CRC, carcinoma in situ (CIS), high risk lesion (HRL) and intermediate risk lesion (IRL)-, and non-CR lesions—including negative (N), lesion not associated to risk (LNAR) and low risk lesion (LRL). Using the colonoscopy information, we selected a representative set of samples for microbiome characterization, aiming for a balanced representation of clinically relevant colonoscopy outcomes. We performed DNA extraction and 16S metabarcoding analysis of the V3-V4 region on the selected samples (see Materials and Methods, Section 2.2). A total of 2889 FIT-positive samples passed all quality filters and were included in the study (see Materials and Methods, Section 2.3). A summary of the distribution of these samples across several characteristics is shown in Table S2. We obtained a mean value of 56,219.03 filtered reads per sample, which comprised a total of 376 assigned taxa. Bacteroidetes and Firmicutes were the most represented phyla, and the ten most abundant genera were, in this order: *Bacteroides*, *Faecalibacterium*, *Prevotella*, *Blautia*, F.Lachnospiraceae.UCG, *Ruminococcus*, *Agathobacter*, *Bifidobacterium*, *Alistipes* and *Akkermansia* (Figure S1). These results are consistent with previous studies using stool samples [39,40,41,42,43], and with earlier analyses showing a high correspondence between stool and FIT samples from the same individuals [36,37]. We compared our data with that of a recent Spanish population gut microbiome study [27]. The two cohorts differ in several features such as the age range, but most notably our cohort was entirely formed by individuals with blood in stool, a factor shown to impact the gut microbiome [44], and hence differences are expected. Nevertheless, the two sample sets were largely similar in terms of dominating phyla and genera, reinforcing the validity of FIT sampling as a proxy of the gut microbiome (Figure S2). 

### 3.2. Changes in Microbiome Composition along the Path from Healthy Colon to Colorectal Cancer

We quantified the overall microbiome diversity by computing alpha and beta diversity metrics. We only observed significant differences (Kruskal-Wallis, *p* < 0.05) in the observed index alpha diversity metric (which measures the number of species per sample), and in the Simpson index (which considers taxa abundances) when considering all diagnoses, but not when specifically comparing clinically relevant (CR) vs. non-CR samples (Figure S3). For the Shannon and Simpson indices, which consider differences in taxa abundances, we only observed significant differences with the Simpson index (which assigns more weight to dominant species) when considering all diagnoses. We produced multidimensional scaling (MDS) plots using distances between the microbial profiles of samples (beta diversity) such as the Aitchison distance (Figure S4). We did not observe a clear clustering of samples with the same diagnosis or risk (CR vs. non-CR). However, with the adonis test and Aitchison distance, we detected a significant effect of the diagnosis (*p* = 0.001) considering sex and age as covariates, and the sequencing run as a possible source of batch effect. The ANOSIM test also supported significant differences between the diagnostic groups and a higher similarity within groups (R: 0.07463, *p*-value: 0.001). Altogether, these results suggest the existence of significant but subtle differences in the overall microbiome composition between FIT-positive samples with different colonoscopy outcomes. 

**Figure 1:**
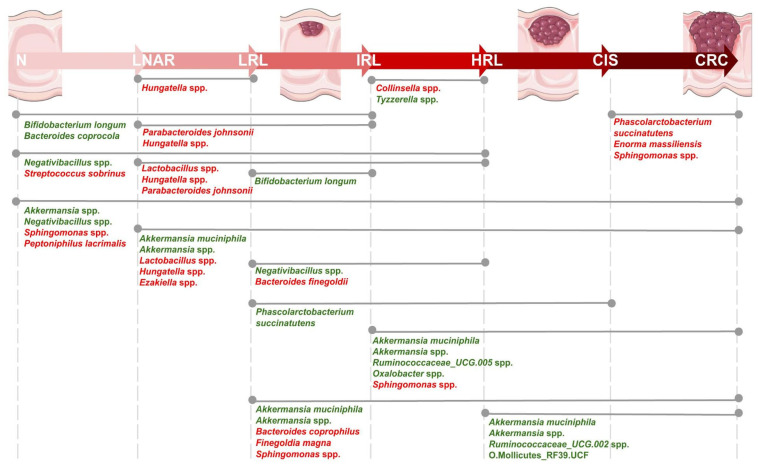
Representation of the 34 bacterial species found as significantly differentially abundant in pairwise comparisons of diagnoses following the path from healthy colon to colorectal cancer (Tukey test, p.adjusted < 0.05, n = 2565). Different colonoscopy diagnoses are depicted from left to right following this path, with healthier states at the left and in the following order: N, negative; LNAR, lesion not associated to risk; LRL, low risk lesion; IRL, intermediate risk lesion; HRL, high risk lesion; CIS, carcinoma in situ; CRC, colorectal cancer. Lines connecting different diagnoses indicate comparisons, with differentially abundant species names indicated. Colors in the species names indicate the direction of the change with red indicating decrease and green increased relative abundance with respect to the healthier state.

We next used comparative analysis to detect significant differences in the relative abundance of taxa according to the variables considered (Table S3). These analyses identified 34 species whose abundance varied significantly across colonoscopy diagnosis (Data S3 and Figure 1). 

**Figure 2:**
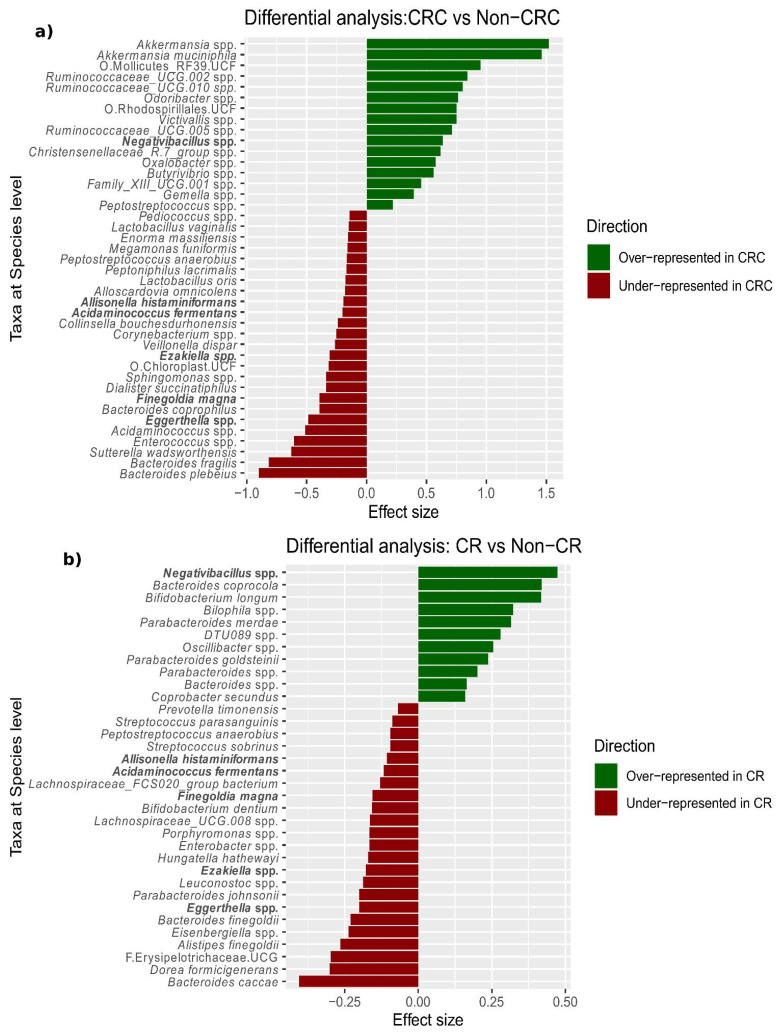
The effect size of species found as significantly differentially abundant when comparing CRC vs. non-CRC samples (n = 2565) (**a**) and CR vs. non-CR samples (**b**). Bars are green for overrepresentation and red for underrepresentation. The bars are sorted according to the effect size. In bold are the highlighted taxa that appeared as differentially abundant in both comparisons.

Based on the observation that CRC was the diagnosis with the most distinct microbiome (Figure 1), we specifically compared CRC to non-CRC samples, which revealed 41 differentially abundant species (Figure 2a and Data S4). These included overrepresentation of *Akkermansia muciniphila* and *Akkermansia* spp., as well as underrepresentation of *Bacteroides plebeius* and *Bacteroides fragilis* in CRC compared to non-CRC samples. In addition, we found that the ratio between species abundance (balance) most associated with CRC-status was given by a decrease (as compared to non-CRC samples) in a group of taxa comprising *B. fragilis* (G1: *Bifidobacterium* spp., *Bacteroides fragilis*, *Sutterella wadsworthensis*, and *Eggerthella* spp.), with respect to a second group of taxa including *Akkermansia* spp. (G2: *Akkermansia* spp., *Gemella* spp., *Peptostreptococcus stomatis*, *Adlercreutzia* spp. and *Butyrivibrio* spp.). We explored the progression of the levels of *Akkermansia* genus along the path from normal colon to CRC, observing an increase from HRL to carcinoma in situ and from carcinoma in situ to CRC. (Figure S5). 

Finally, we applied the same linear model to the comparison of CR vs. non-CR samples, which identified 34 differentially abundant species, of which six were shared with the comparison above (Figure 2b and Data S5).

We next explored whether changes in the microbiome correlated with other variables collected in the study such as the number of polyps observed in the colonoscopy examination and lifestyle parameters collected by a questionnaire. Colorectal polyps, which are benign tumors that project onto the colon mucus and protrude into intestinal lumen [45], have long been identified as potential precursors of CRC. Polyp size, localization and histology, among other factors, may influence their role in CRC development. Our study includes the information of the presence or absence of polyps, wherein colonoscopy detected the presence of polyps in 66.82% of samples, with the numbers of polyps ranging from one to 22. We observed that some CRC (32/134, 23.88%) samples had no polyps, whereas some negative samples had from 1 to 3 polyps (21/925, 2.27%), and some lesions that were not associated with a clinically relevant colonoscopy had a considerable amount of polyps (from 1 to 11 polyps, e.g., two individuals diagnosed by LNAR and LRL had 11 polyps). We searched for species whose abundance correlated significantly with the number of polyps and found 33 such cases (Data S6), including *B. vulgatus,* which was associated with systemic inflammation and CRC progression [46]. Finally, we found no significant effect of the CRC tumor stage on the microbiome composition, although this may relate to limited sample size (n = 101, Adonis test, R2: 0.03104 *p* value: 0.386). A subset of the included individuals (n = 2016) responded to a lifestyle questionnaire. We assessed the impact of different variables on microbiome composition, and found a significant impact of weight, height, regular exercise, smoking, alcohol, vegetables and processed meat intake and anti-inflammatory drug use, as observed in previous studies. When this impact was considered in conjunction with the diagnosis, we observed only a significant effect of the vegetable’s intake (Figure S6). 

### 3.3. Diagnosis-Specific Co-Occurrence and Functional Profiles

**Figure 3:**
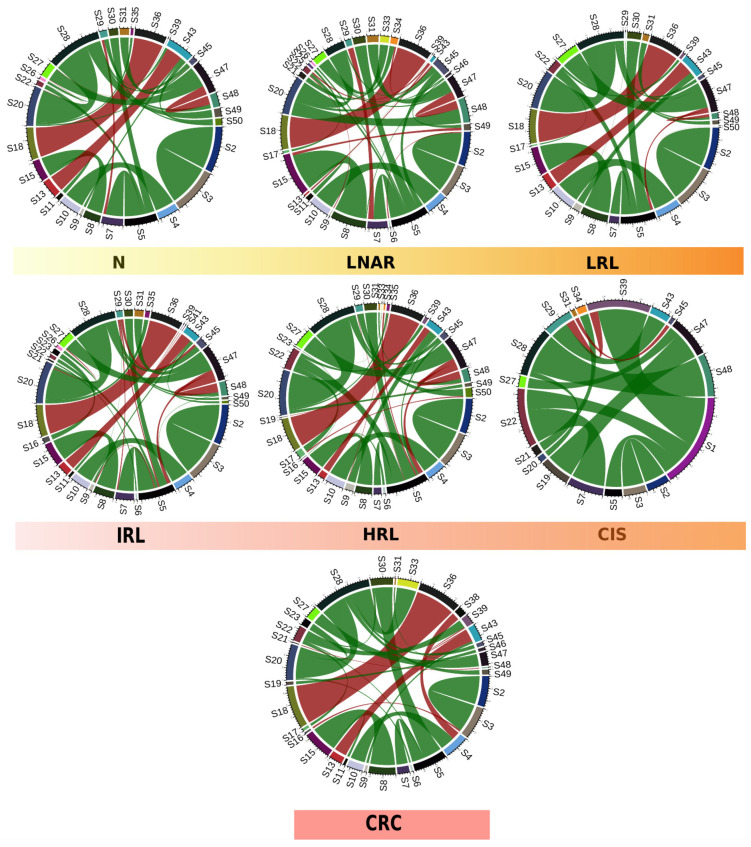
Circos plots representing the correlation weight matrices obtained from the computed networks of co-occurrence according to the diagnosis (negative (N) n = 925, lesion not associated to risk (LNAR) n = 90, low risk lesion (LRL) n = 681, intermediate risk lesion (IRL) n = 638, high risk lesion (HRL) n = 397, carcinoma in situ (CIS) n = 24, and colorectal cancer (CRC) n = 134) considering the top 50 taxa. The green connections are for positively correlated and the red connections are for negatively correlated taxa. The thickness of the arrows represents the strength of the correlations.

To gain further insights into the changes of microbial composition along the path from healthy tissue to CRC, we used proxies for community interactions (co-occurrence networks), and functional potential (functional inference from taxonomic assignment). We first built species networks showing patterns of correlated abundances for samples with each specific diagnosis and compared them (see Materials and Methods, Section 2.5). By constructing and representing co-occurrence networks based on the 50 most abundant taxa, we qualitatively observed differences across the diagnoses along the path from healthy colon to CRC (Figure 3). These differences were confirmed by computing hamming distances between co-occurrence networks of successive pairs of diagnoses along this path: 0.024 (N vs. LNAR), 0.023 (LNAR vs. LRL), 0.014 (LRL vs. IRL), 0.016 (IRL vs. HRL), 0.030 (HRL vs. CIS) and 0.028 (CIS vs. CRC). According to this, the last two steps in the progression from healthy tissue towards CRC (HRL to CIS and CIS to CRC) display the largest dissimilarities. Similar results were obtained using an alternative approach based on Spearman correlations: 66% (N vs. LNAR), 65% (LNAR vs. LRL), 53% (LRL vs. IRL), 53% (IRL vs. HRL), 79% (HRL vs. CIS) and 73% (CIS vs. CRC). 

S1: *Bacteroides vulgatus*, S2: *Akkermansia muciniphila*, S3: *Akkermansia* spp., S4: *Collinsella aerofaciens*, S5: *Bacteroides* spp., S6: *Agathobacter* spp., S7: *Bacteroides uniformis*, S8: *Faecalibacterium prausnitzii*, S9: *Holdemanella* spp., S10: *Collinsella* spp., S11: *Faecalibacterium_CM04-06* spp., S12: *Ruminococcus bromii*, S13: *Erysipelotrichaceae_UCG-003* spp., S14: *Escherichia* spp., S15: *Faecalibacterium* spp., S16: *Dorea longicatena*, S17: *Alistipes putredinis*, S18: *Phascolarctobacterium* spp., S19: *Ruminococcus* spp., S20: *Blautia* spp., S21: *Subdoligranulum* spp., S22: *Alistipes* spp., S23: *Dorea* spp., S24: *Bifidobacterium* spp., S25: *Bacteroides massiliensis*, S26: *Streptococcus* spp., S27: *Ruminococcaceae_UCG-002* spp., S28: *F.Lachnospiraceae.UCG*, S29: *Prevotella* spp., S30: *Parabacteroides* spp., S31: *Ruminococcaceae_UCG-014* spp., S32: *Prevotellaceae_NK3B31_group* spp., S33: *F.Ruminococcaceae.UCG*, S34: *Coprococcus* spp., S35: *Anaerostipes* spp., S36: *Dialister* spp., S37: *Roseburia* spp., S38: *Lachnospira* spp., S39: *Barnesiella* spp., S40: *Bacteroides coprocola*, S41: *F.Muribaculaceae.UCG*, S42: *Paraprevotella* spp., S43: *Catenibacterium* spp., S44: *O.Rhodospirillales.UCF*, S45: *Erysipelatoclostridium* spp., S46: *Lachnospiraceae_NK4A136_group* spp., S47: *Christensenellaceae_R-7_group* spp., S48: *Lachnoclostridium* spp., S49: *Ruminiclostridium* spp. and S50: *Alloprevotella* spp.

Of note, some of the specific differences that we detected were that *Akkermansia muciniphila* and *Akkermansia* spp. were found as positively correlated in all the diagnoses, but only in CRC we observed a negative correlation of the *Akkermansia* spp. and *Dorea longicatena* species. In contrast, in LNAR and LRL we found a negative correlation of these species with *Agathobacter* spp. and *Alloprevotella* spp., respectively. Also, we observed a positive correlation between *Collinsella aerofaciens* and *Collinsella* spp. in all the diagnoses except in the CIS group, and only in CRC we observed a negative correlation with another taxon, *Lachnospira* spp. 

**Figure 4:**
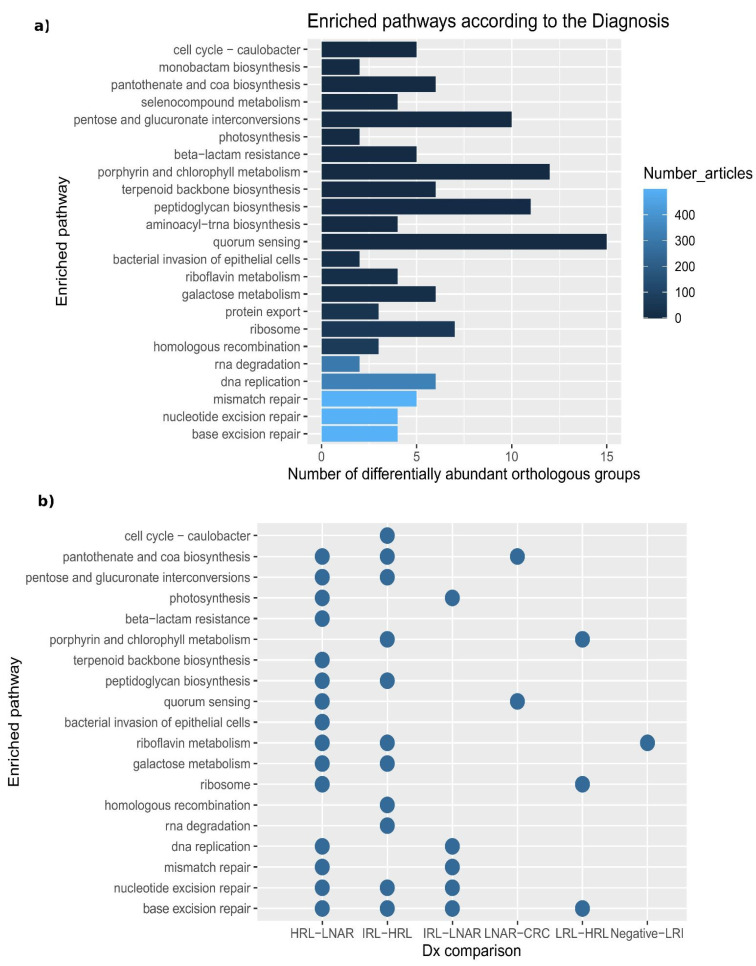
Enriched pathways according to the diagnosis. (**a**) The length of the bar indicates the number of differentially abundant OGs involved. The bars are sorted and colored according to the number of articles for which a given pathway has been linked to CRC. (**b**) Dotplot representing the pairs of diagnoses in which we found differentially abundant OGs involved in the enriched pathways. Of note, monobactam biosynthesis, protein export, selenocompound metabolism and aminoacyl-trna biosynthesis are not represented because multiple comparison tests did not detect involved OG as differentially abundant in any pairwise comparison.

Co-occurrence networks may reflect underlying microbial communities that may interact metabolically. To obtain functional insights we inferred the functional potential of the microbiota in each sample by exploring metabolic pathways and processes associated with 2927 orthologous groups (OG, i.e., functionally-annotated gene families) in 376 taxa present in our samples (see Materials and Methods, Section 2.6). By studying the variation of abundance of OGs across samples, we identified 184 that were significantly differentially abundant according to the diagnosis (Data S7). The differentially abundant OGs were linked to 23 enriched pathways (containing more than 10 predicted OGs and 10% or more differentially abundant OGs involved), many of which have been linked to CRC in the literature, according to a text-mining approach (Figure 4a).

When performing pairwise comparisons between diagnoses along the path from healthy colon to CRC, we only observed significant differences of OGs in the transition from IRL to HRL (Figure 4b and Data S8). For instance, some of the OGs that we found as significantly differentially abundant between these two diagnoses were: K00850, K00963, K02231, which are involved, respectively, in galactose metabolism, RNA degradation, pentose and glucuronate interconversions, porphyrin and chlorophyll metabolism, peptidoglycan biosynthesis and cell cycle—Caulobacter. 

### 3.4. Development of a Two-Phase Machine Learning Classifier

**Figure 5:**
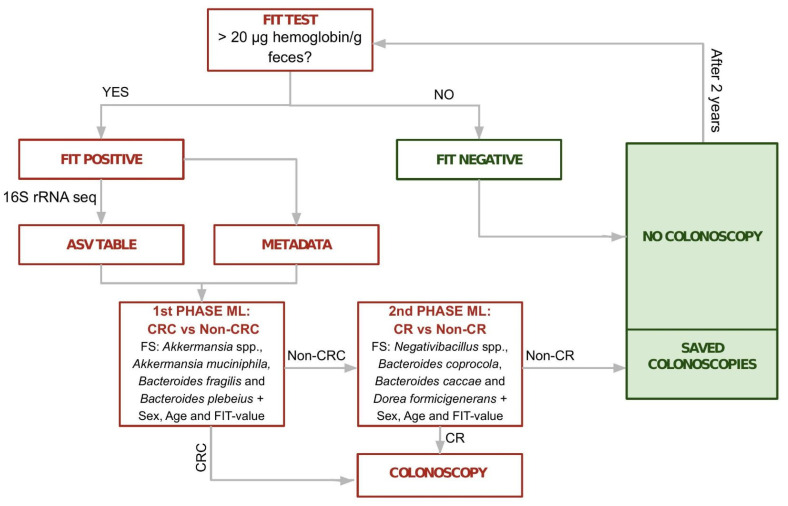
Flow chart of the proposed methodology (4-4 taxa classifier). FIT positive samples are subjected to microbiome profiling by 16S rRNA gene sequencing. Then a two phase classifier is applied. First the algorithm classifies CRC vs. non-CRC samples. Samples that are classified as non-CRC in the first phase are subjected to a second model that classifies CR vs. non-CR samples. FIT: fecal immunochemical test; CRC: colorectal cancer, CR: clinically relevant.

The observed differences in bacterial composition across samples with varying diagnoses suggest a diagnostic potential for the microbial compositions of FIT-positive samples that could be harnessed to improve the efficiency of current screening programs. With the aim of reducing unnecessary colonoscopies while maintaining a high sensitivity, we explored machine learning approaches to develop a sample classifier able to discriminate samples with clinically-relevant diagnoses (CR, CRC samples and lesions of higher risk). Contrary to most automated classifiers that aim at maximizing accuracy, we intentionally put our focus on achieving high sensitivity at the cost of reduced accuracy. This is justified because, in a clinical context, false negatives (i.e., persons with clinically relevant lesions that do not proceed to colonoscopy) are of higher medical concern as compared to false positives (persons with no lesions that undergo colonoscopy), and because the main aim was to reduce the already high level of false positives in current FIT-based screenings without increasing the amount of false negatives. To derive this predictor, we explored the effect of using different machine learning algorithms, and the use of feature selection to restrict the parameter set to all bacterial taxa showing significant differences, or to a subset of them (see Materials and Methods, Section 2.7). When including more taxa, we observed a better area under the curve (AUC) and specificity (Table S4) This fact can be translated to better reduction of false-positive rates. On the other hand, when restricting to only a panel of taxa, we obtained better recall and sensitivity for CRC and CR samples but poor AUC and specificity (Table 1). However, in the context of the current screening, there is still a satisfactory reduction of the false-positive rate with a good prioritization of relevant cases. We achieved optimal results, in terms of inclusion of clinically relevant samples, with a two-phase classifier trained to classify CRC samples in a first phase and CR samples in a second phase. This final classifier considered information on sex, age and fecal hemoglobin concentration, and abundances from two different subsets of four taxa (first phase: *Akkermansia* spp., *Akkermansia muciniphila, Bacteroides fragilis* and *Bacteroides plebeius* and second phase: *Negativibacillus* spp., *Bacteroides coprocola*, *Bacteroides caccae* and *Dorea formicigenerans*) (Figure 5). This classifier obtained an average 98.98% sensitivity for CRC samples and 97.98% for clinically relevant samples (Table 1B). 

We validated our strategy on two independent datasets. We first constructed a model with all the samples (without including *Bacteroides fragilis*, see Materials and Methods, Section 2.7) and tested it on an independent cohort of 135 FIT-positive samples from the USA [37]. The results of this adjusted model in the USA cohort yielded 100% sensitivity for CRC and 98.46% for CR lesions, reducing 20 % of the unnecessary colonoscopies (Table S5A). We also performed an additional validation, in this case including both 4-4 taxa panels, with an independent dataset composed of 100 additional samples from the same Catalan screening detecting all CRC samples, 96% of the CR samples and having a reduction of 12% of the false positives (Table S5B). This last test set was balanced, and it was used for further optimization of the classifier. The corresponding ROC curves are represented at (Figure S7). 

We explored how changing some parameters of the classifier affected sensitivity and the number of saved colonoscopies. For instance, by penalizing less the minority class (CR) at the second phase, we obtained better reduction of unnecessary colonoscopies (26%) but at the cost of including less CR samples (90%). Similarly, the number of samples to be tested for the microbial signature can be reduced by applying a FIT-value threshold above which a benefit of colonoscopy is assumed. For instance, applying a value of 954 μg hemoglobin/g feces (3rd quartile in our CR samples) for such a threshold, which is passed by 18% of our samples, would save 14% of unnecessary colonoscopies at the end of the process and reduce the need for microbiome testing. When we combined both approaches, we could reach 30% of saved colonoscopies, at the cost of a reduction of CR detection (87%). However, in all the mentioned cases we detected 100% of the CRC samples. This shows that our algorithm can be fine-tuned to optimize cost-effectiveness (Figure S8). A comparison of our algorithm with the current FIT strategy and other available solutions (GoodGut [47] and ColoGuard [48]) revealed higher sensitivity for both CRC and CR while maintaining a significant reduction of the current false positive rate and, importantly, without the need of collecting a separate sample from the screening (Table S6).

We next assessed possible alternative subsets of species included in the lists of differentially abundant taxa according to the diagnosis (Data S4 and S5) as potential features for the classification (See Materials and Methods, Section 2.7). We tested a total of 948 models and selected 13.5% of them (128/948). The strategy that led to more selected models was the one including subsets of 4 taxa with highest effect size, selecting half of the trained models (Figure S9A). The two *Akkermansia* species were the taxa that were most often included in selected models (Figure S9B) and 96.88% of the selected models included at least one of the 8 taxa used as features in the 4-4 taxa panel classifier (*Akkermansia muciniphila*, *Akkermansia* spp., *Bacteroides fragilis*, *Bacteroides plebeius*, *Bacteroides coprocola*, *Negativibacillus* spp., *Dorea formicigenerans* or *Bacteroides caccae*). These results suggest that different combinations of biomarkers drawn from the identified differentially abundant taxa can effectively be used to classify samples according to their clinical relevance. 

## 4. Discussion

CRC is a healthcare challenge and one of the leading causes of cancer-related deaths worldwide [49]. Early diagnosis of CRC is key for efficient treatment and for the survival of the patients and, hence, there is a strong interest in implementing diagnostic screenings for populations at risk. Colonoscopy, which is the gold standard for CRC diagnosis, is an expensive, time-consuming, invasive technique with potential complications. To minimize the use of colonoscopy only to cases that are more likely to benefit, population screening programs use less specific, non-invasive tests to pre-screen for risk of CRC. Immunochemical methods, such as FIT, have commonly been used as pre-colonoscopy tests in a two-step approach [50], but they have high false-positive rates, which results in unnecessary colonoscopies. This, in turn, increases healthcare costs and saturates endoscopy units, limiting the efficiency of population screenings. Considering this, there is a need to reduce the false-positive rate of the initial screening step by identifying new biomarkers and developing new risk scores. In this context, the gut microbiome has been suggested as a promising source for biomarkers with diagnostic potential in CRC [51]. In this project, we set out to investigate the potential of FIT samples to identify diagnostic markers and changes in the microbiota along the path from healthy colonic tissue to CRC. 

Recent studies have shown the potential of the gut microbiome for CRC screening but these are mainly based on other types of samples [8,41,52,53] (e.g.,: gFOBT or stool samples) and are often focused on the comparison of CRC and healthy controls. In contrast, the focus of this project was on improving current screening programs based on FIT testing, using material from the same samples, and focusing on distinguishing clinically relevant cases (not only CRC) from FIT-positive samples (not the usual healthy baseline but the baseline of the population currently sent for colonoscopy). 

Our results support the use of sampled material directly from FIT containers for microbiome analysis, avoiding the complex and costly collection and processing of separate stool samples that are widely and traditionally used to represent the gut microbiome [36]. More importantly, we show that the collected fecal material was enough to perform both the hemoglobin analysis and DNA extraction, and that the DNA was of sufficient quantity and quality to efficiently perform 16S metabarcoding. Earlier studies have also shown good conservation of DNA from frozen samples and close correspondence between microbiome profiles obtained from FIT samples and matching fecal material [54,55]. This is consistent with our results, which showed that the identified taxa and abundances are typically found in studies that use stool samples although observing differences that can be attributed to cohort or methodological particularities. Hence, our study shows that we can use the same fecal sample for both FIT and microbiome analyses, facilitating the implementation of microbiome-based biomarkers in currently ongoing population screening programs. It is well known that a high percentage of CRCs emerge from premalignant polypoid lesions (i.e., adenomas and serrated lesions), which progress to CRC following a multi-stage development driven by both genetic and environmental risk factors [56]. Diet and lifestyle are key environmental factors associated with the presence of adenomas and their progression to CRC, likely through alterations of the gut microbiome. In our study, we captured differences between the fecal microbiome profiles along the various stages in the path from normal colonic epithelium to CRC. To the best of our knowledge, this is the first large microbiome study considering such a detailed and rigorous diagnostic classification associated with the included samples, which comprises different lesions in addition to healthy and CRC samples (Table S1). We did not observe disparate overall microbiome compositions between different clinical diagnoses but did find significant changes in particular taxa. Thus, different combinations of small but relevant changes may drive microbiome influence on CRC progression. In addition, it must be considered that microbiota alterations might more profoundly affect lesions and surrounding tissues, which may result in only subtle differences in the overall composition of the fecal material contained within FIT tubes. 

Expectedly, we observed that CRC was the diagnostic group that had the most distinct microbiome profile. Taxa with the highest deviations in CRC-associated samples were *Akkermansia muciniphila* and an unclassified species from the same genus (*Akkermansia* spp.), which were overrepresented in CRC compared to the other samples, and *Bacteroides fragilis* and *Bacteroides plebeius*, which were underrepresented. Of note, *A. muciniphila* is a mucin-degrading bacterium and mucins such as MUC1 and MUC5AC are known to be overexpressed in CRC patients [57]. Hence, an increase of substrate availability could influence the observed higher abundance of this species. Interestingly, it is known that if microorganisms or their products cross the host epithelial barrier, both the immune and mesenchymal defenses respond with a signaling cascade (e.g., activation of NF-kB and STAT3) in order to maintain epithelial integrity. This fact has a selective impact on the gut microbiome and triggers mucin and antimicrobial peptide secretion [58]. *A. muciniphila* was found as overrepresented in other populations, and it was recently claimed as a potential biomarker for CRC in tissue [59]. 

Contrary to other studies with fecal and tissue samples that reported an enrichment of *Bacteroides fragilis* in CRC [58], we found this species to be underrepresented in these samples. Previous studies suggested that *B. fragilis* plays a key role in the development of CRC through the action of its toxin (BFT), which can influence colorectal tumorigenesis by disturbance or activation of signaling pathways that produce chronic intestinal inflammation and tissue injury [60]. However, we found this underrepresentation comparing CRC vs. non-CRC (including all the adenomas), as opposed to these other studies which compared CRC vs. healthy samples. Previous studies have shown that there are different strains of *B. fragilis* along the gastrointestinal tract apart from the mentioned BFT-producing strains, such as a non-toxigenic *B. fragilis* which has an immunogenic capsular component, and the Polysaccharide A that promotes mucosal immune development and whose increase has not been associated to CRC [61,62]. 

We also observed an influence on the differences of the microbiome driven by variables like sex and age and, interestingly, by the number of polyps. As mentioned above, the presence of polyps can be a sign of risk to development or progression of CRC, so the study of the microbiome associated with polyps can serve as a source of predictive biomarkers for CRC. Some of the genera whose abundance correlated with the number of polyps were also reported in previous studies in relation to risk for CRC polyps (e.g., *Bacteroides*, *Blautia* and *Bifidobacterium).* However, the presence of polyps does not necessarily lead to the development of CRC and some patients with particular genetic profiles may present numerous polyps [63].

It is known that the presence of certain metabolites, DNA damage, and inflammation are all factors driving CRC progression [64]. Changes in the microbial composition or functionalities can promote a more optimal microenvironment for the development of CRC. Conversely, CRC progression can alter the surrounding environment and therefore affect microbial communities. In our study, we inferred the potential functionalities of the microbiome profiles associated with each colonoscopy outcome and observed OGs that were significantly differentially abundant across diagnoses. Interestingly, we observed that the transition from intermediate risk lesion to high risk lesion was the stage with the greatest alteration of functional and metabolic capacities. Some examples of enriched pathways were galactose metabolism, RNA degradation, pentose and glucuronate interconversions and quorum sensing. In this regard, it has been reported that microbes can interact with cancer cells through their quorum sensing peptides and influence metastasis [65]. Also of note, many of the pathways found are related to DNA repair. This may reflect a toxic environment for the microbial DNA, perhaps caused by the bacterial metabolism. This same environment could be damaging to the host DNA, supporting a genotoxic pathway connecting the microbiome and CRC development [66]. Our results are based on 16S rRNA sequencing, which is a cost-effective approach that can be applied to many samples. In particular, the presented results related to functional inference should be confirmed using shotgun metagenomics or meta-transcriptomics approaches, which will provide better resolution. However, previous studies demonstrated high correlation of functional profiles predicted from 16S rRNA sequencing data and from metagenomes [67], and we believe the data presented here are a good proxy for generating testable hypotheses. 

Metabolic capacities of some microorganisms, such as the mentioned mucin utilization of *A.muciniphila*, can result in sources of nutrients or energy for other microbes in the gut. The study of correlated abundances between different microbes is interesting in this context. We detected distinct taxon co-occurrence patterns in the studied diagnoses that likely reflect changes in microbial ecosystems and their metabolic interactions that accompany the transitions towards CRC development. It is interesting to account for these patterns because of the dominant functional redundancy of the gut microbiome: some bacteria can share functions and exert similar influences on the development and progression of CRC. In addition, it is still unclear how microbes modulate each other, or how they shape the immune environment of the tumor, and these co-occurrence patterns can shed light in this direction [58]. For instance, we observed an exclusive negative association between *Dorea longicatena* and *Akkermansia* spp. only in the CRC group. 

The presented machine learning prediction results show a potential role of the microbial composition of FIT samples in CRC screening. We derived a two-phase classifier with high sensitivity for CRC and other CR samples with a small but significant reduction of the false positive rate. In the context of the Barcelona screening program [13], in which there is an average participation of 50%, approximately 5% of participants have a positive FIT result. Of them, around 3–5% have CRC detected during colonoscopy and an additional 30% have a CR lesion associated with CRC risk requiring a more intensive surveillance, whereas around 65% have a normal colonoscopy or only non-CR lesions are detected. Therefore, translating our results to this clinical context and considering the mean participation and diagnosis obtained during the last four available rounds, we would save a range between 423 (12%) to 1057 (30%) unnecessary colonoscopies each year, while maximizing the inclusion of CR individuals (Data S9). 

By reducing the number of unnecessary colonoscopies and increasing cost-effectiveness of current population screenings, microbiome-based tests such as the one explored here, could not only save money and time but also increase participation and adherence rates. The present study has some limitations, such as the imbalance in some of the diagnoses, and the lack of more detailed information on polyps or lesion characteristics (e.g., localization, size, histology), genetic profiles, or past treatments, which can be factors influencing the microbiome. However, this lack of information, which is difficult to access beforehand, is also a strength of our study, showing that with just the FIT sample and information on the sex and age of individuals we can draw some conclusions and obtain a classification of the samples with high sensitivity for CRC and CR samples. Further studies are necessary to validate these findings in different cohorts and to properly assess cost-effectiveness in the framework of a health economics analysis that considers direct and indirect costs of colonoscopy and microbiome analysis from FIT samples. Finally, further developments such as a targeted quantification of a species panel by multiplex PCR, or implementations in the FIT tube to accommodate this additional test, will likely further reduce costs and facilitate the adoption of microbiome-based tests. 

## 5. Conclusions

Colorectal cancer (CRC) is a leading cause of cancer deaths worldwide with a substantial challenge in its diagnosis, which if done early could improve overall survival. Our study suggests a potential role of the microbiome in the path from normal epithelia to CRC, revealing taxa, metabolic features and co-occurrence changes along this progression. The proposed classifier and its possible cost-effectivity optimization as well as the addition of other layers of information or current in-use clinical biomarkers such as microRNAs, gene mutations and DNA methylation, that are already stated as potential biomarkers, can be a potential tool for clinical proposes and improvement of current CRC screening.

### Patents

A patent covering the use of microbial biomarkers for CRC and CR detection published in this manuscript has been filed. 

## Figures and Tables

**Table 1:** Performance of the two-phase machine learning predictor. The reported values are mean values obtained from the 100 random splits and include a panel of four taxa for each of the phases plus sex, age and FIT-value. Samples with missing metadata were discarded from this analysis (n = 2817). (A) Average of area under the curve (AUC), recall and specificity for each of the phases. (B) Average sensitivity for clinically relevant samples and for each of the diagnoses included in this group.

**(A)**			
	**AUC**	**Recall**	**Specificity**
**FIRST PHASE**	0.565368	0.8709974	0.2597385
**SECOND PHASE**	0.5358411	0.8052662	0.2664159
**(B)**			
	**Average sensitivity (%)**		
CR *	97.98		
**IRL**	97.71		
**HRL**	98.06		
**CIS**	98.54		
**CRC**	98.98		

* The average CR sensitivity re-proportionated according to the population (data from the Barcelona colorectal cancer screening, presented at Data S9) is 98.05%.

## Data Availability

The dataset supporting the conclusions of this article is available in the NCBI Sequence Read Archive (SRA) under the BioProject ID PRJNA792716. The study relies on open source software listed in the methodology.

## Supplementary Materials

The following supporting information can be downloaded at: https://www.mdpi.com/article/10.3390/cancers15010120/s1, Supplementary Data (large tables): Data S1. Strengthening The Organization and Reporting of Microbiome Studies (STORMS) checklist; Data S2. Metadata will be available prior to publication; Data S3. Table of the taxa at the species level that we found as differentially abundant according to each of the fixed effects included in the linear model. Only significant *p*-values are reported. Samples with missing metadata were not considered in this analysis, (n = 2565); Data S4. Table of the taxa at the species level that we found as differentially abundant according to each of the fixed effects included in the linear model when comparing CRC vs. non-CRC. Only significant *p*-values are reported. Samples with missing metadata were not considered in this analysis, (n = 2565); Data S5. Table of the taxa at the species level that we found as differentially abundant according to each of the fixed effects included in the linear model when comparing clinically relevant (CR) vs. non-Clinically relevant (non-CR) samples. Only significant p-values are reported. Samples with missing metadata were not considered in this analysis, (n = 2565); Data S6. Table of species found as differentially abundant according to the number of polyps, and the significance values (*p*-value < 0.05). Samples with missing metadata were not considered in this analysis; (n = 2565); Data S7. List of differentially abundant OG according to the diagnosis and the significance values (*p*-value < 0.05); Data S8. Summary of the significant results obtained when applying multiple comparisons between diagnoses. Significant *p* values are reported (Tukey test, p.adjusted < 0.05). The *p*-value has the sign of the corresponding effect size, indicating the direction of the difference; Data S9. Statistics of the last four available rounds of results from the Catalan CRC screening in Barcelona; Supplementary Material (Figures and small tables): Figure S1. Pie chart representing the 10 most abundant genera of studied CRIPREV samples. The other genera were grouped and named as “others”; Figure S2. Comparison of FIT positive 16S samples from the present study and stool 16S samples from an independent study. (A) Multidimensional scaling plot (MDS) representing the Aitchison distance and Shannon index according to the source project. (B) Barplot representing the present phyla. Each column represents a sample; Figure S3. Alpha diversity characterization, (n = 2889). The lines inside the boxplots represent the medians for each of the groups. Statistical test: Kruskall-Wallis or Wilcoxon test, with a significant result when *p* < 0.05. (A) Observed index according to the diagnosis (carcinoma in situ (CIS), colorectal cancer (CRC), lesion that is not associated to risk (LNAR), high risk lesion (HRL), low risk lesion (LRL), intermediate risk lesion (IRL) or negative (N) samples) and risk (clinically relevant (CR) vs. non-clinically relevant (non-CR) samples) variables. (B) Shannon and Simpson indices according to the diagnosis; Figure S4. MDS plots using Aitchison distance, (n = 2889). The samples are colored according to the diagnosis. 95% confidence ellipses are represented for each of the diagnosed groups; Figure S5. Box plot of the Akkermansia clr according to the different explored diagnosis, (n = 2889). Negative (N), lesion not associated to risk (LNAR), low risk lesion (LRL), intermediate risk lesion (IRL), high risk lesion (HRL), carcinoma in situ (CIS) and colorectal cancer (CRC); Figure S6. Summary of the results of the adonis test, evaluating the effect of lifestyle variables on the overall composition. Only significant (*p*-value < 0.05) results are colored, including the p-value in each of the cells. The assessment of the individual effect of each variable is in the orange column, while is the impact using as covariate the diagnosis is in the pink column. The explained variability (R2) was used for the color intensity of the cells; Figure S7. ROC curves for each of the phases in the different validations performed. First phase: CRC vs. others, Second phase: clinically relevant vs. non-clinically relevant (a) USA cohort, (b) 100 extra samples from the CRC screening; Figure S8. Percentage of saved colonoscopies and clinically relevant sensitivity according to the different specifications of the proposed classifier; All_taxa: All the intersecting taxa between the CRIPREV and the validation datasets were used as features. DA_taxa: All the intersecting differentially abundant taxa between the CRIPREV and the validation datasets were used as features. 4-4 taxa panel: 4 taxa panel for each of the phases. 4-4 taxa panel, adjW: 4 taxa panel for each of the phases, with less penalization of the CR samples in the second phase. FIT_filter_4-4 taxa panel: samples above 954 of the FIT value (μg hemoglobin/g feces) were directed to colonoscopy and the remaining samples were subjected to the classifier. FIT_filter_4-4 taxa panel_adjW: samples above 954 of the FIT value (μg hemoglobin/g feces) were directed to colonoscopy and the remaining samples were subjected to the classifier. Less penalization of the CR samples in the second phase. Figure S9. (A) Potential selection (number of models selected/number of evaluated models, in %) of the different feature selection methods. (B) Average potential selection of each of the 27 studied taxa (number of selected models in which the taxa were included/number of models in which the taxa were included as a feature); Table S1. Criteria and distribution of the colonoscopy-based diagnosis types considered in this project. Columns indicate, in this order: the diagnosis group, the criteria for classification in the group, the number of samples of this study in the given group, and the clinical relevance; Table S2. Characteristics of the included individuals: sex, median and range age and samples deemed of clinical relevance after colonoscopy. * Samples with ‘NA’ value for this parameter are excluded from the calculation; Table S3. Table summarizing differential abundance analysis results considering all the diagnoses following the path from healthy colon to colorectal cancer. We used the linear model: tax_element~diagnosis + hospital + sex + age + n_polyps + FIT_value + (1|run). Samples with missing metadata were not considered in this analysis, (n = 2565); Table S4. Performance of the two-phase machine learning predictor. The reported values are mean values obtained from the 100 random splits. Including 41 and 34 taxa for both phase 1 and phase 2, respectively, plus sex, age and fecal hemoglobin concentration. Samples with missing metadata were discarded from this analysis, (n = 2817). (A) Average of area under the curve (AUC), recall and specificity for each of the phases (B) Average of sensitivity for clinically relevant samples and for each of the diagnosis included in this particular group; Table S5.Performance of the two-phase machine learning predictor on independent datasets. The reported values are obtained by training on all the CriPrev samples (samples with missing metadata were discarded for training the model, n = 2817) and testing on the independent sets. Area under the curve (AUC), recall and specificity for each of the phases and sensitivity for CRC and CR lesions at the end of the two-phase classification were reported. (A) USA cohort. Including a panel of 3 and 4 taxa for phase 1 and 2, respectively, plus sex, age and fecal hemoglobin concentration. (B) 100 extra samples from the Catalan screening; Table S6. Comparison of our algorithm (considering different optimizations, and shadowed cells) with two alternative solutions and the current FIT strategy.

## Appendix A. Authorship Appendix—CRIPREV Consortium

Fundació Institut d’Investigació Biomèdica de Bellvitge (IDIBELL)—ICO

- Josep M Borràs
- Elisabet Guinó, Gemma Ibañez-Sanz, Mireia Obon-Santacana, Ferran Moratalla-Navarro, Ana Diez-Villanueva, Rebeca Sanz-Pamplona, Victor Moreno

Fundació Clínic per a la Recerca Biomèdica (IRS-IDIBAPS)

- Coral Arnau-Collell, Jenifer Muñoz, Josep M Augé, Laia Bonjoch, Anna Serradesanferm, Àngels Pozo, Leticia Moreira, Marcos Díaz-Gay, Sebastià Franch-Expósito, Cristina Herrera-Pariente, Yasmin Soares de Lima, Lorena Moreno, Teresa Ocaña, Sabela Carballal, Ariadna Sánchez, Francesc Balaguer, Jaume Grau, Antoni Castells, Sergi Castellví-Bel
- Elena Asensio, Sara Lahoz, Carolina Parra, Clàudia Galofré, Iván Archilla, Miriam Cuatrecasas, Jordi Camps

Institut Hospital del Mar d’Investigacions Mèdiques (IMIM)

- Joan Gibert, Raquel Longaron, Clara Montagut
- Xavier Bessa, Beatriz Bellosillo, Carme Márquez Márquez, Rebeca Rueda Miret, Rocio Pérez Berbegal, Gabriel Piquer Velaso, Joan Carles Balboa, Ana Cristina Alvarez Urturi, Ines Ana Ibañez Zafon, Sandra Cordero Cerrudo, Miriam Parrilla Carrasco, Bouchra Alouali Moussakhkhar

Institut de Recerca Biomèdica de Barcelona (IRB)

- Toni Gabaldón, Ester Saus, Olfat Khannous-Lleiffe

Fundació Institut d’Investigació en Ciències de la Salut Germans Trias i Pujol

- Sergio Alonso, Beatriz González, Maria Navarro-Jiménez, Andreu Alibés
- Mar Muñoz, Berta Martin, Miguel A. Peinado
