# Supplementary material for: Microbiome Profiling from Fecal Immunochemical Test Reveals Microbial Signatures with Potential for Colorectal Cancer Screening
Source: Cancers (Basel). 2022 Dec 25;15(1):120. doi: 10.3390/cancers15010120 (PMC9817783; doi:10.3390/cancers15010120)
Supplement: Supplementary file 1 [file cancers-15-00120-s001.zip › Supplementary_Material_revised.docx]

**SUPPLEMENTARY FIGURES**


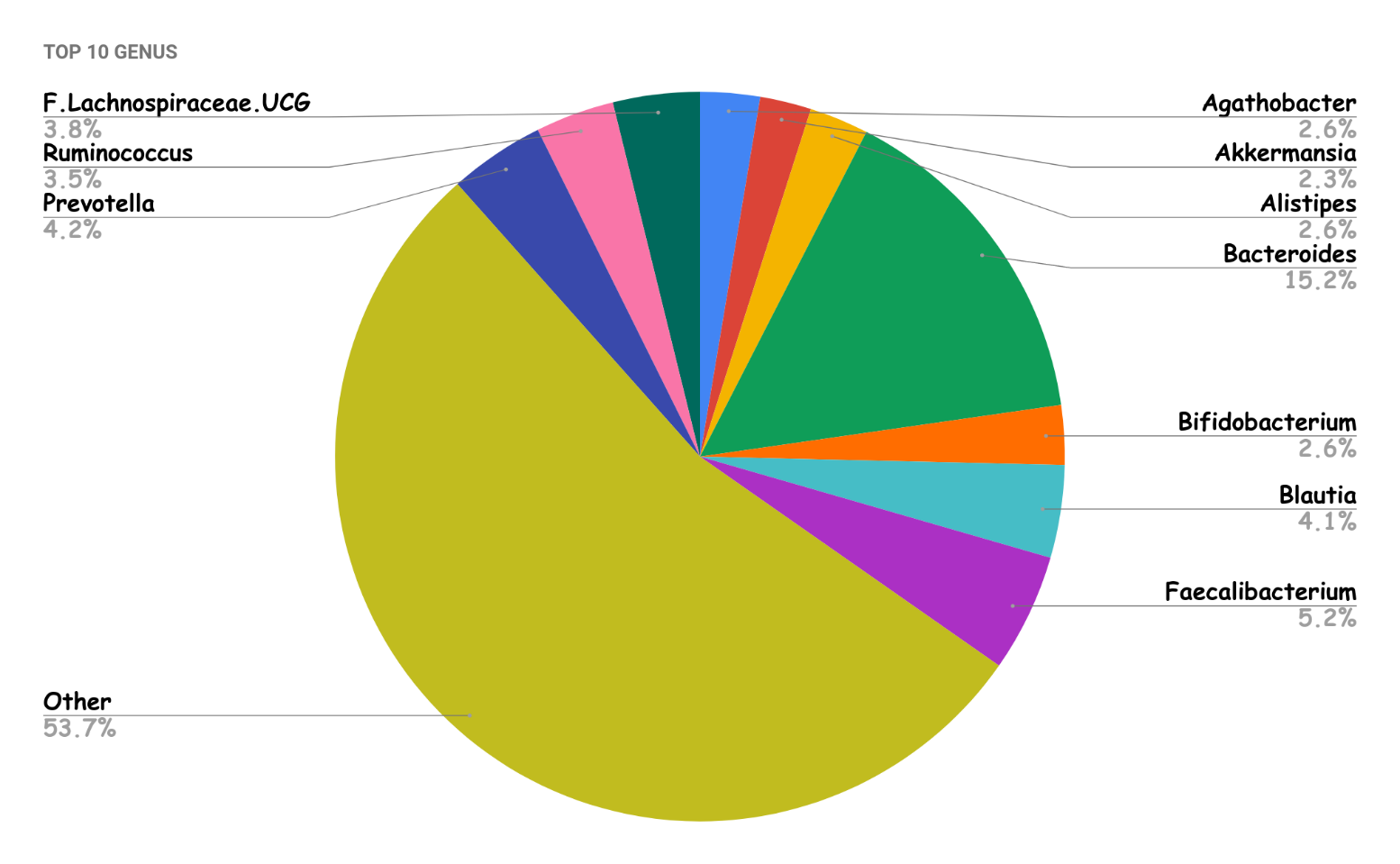


**Figure S1.**  Pie chart representing the 10 most abundant genera of studied CRIPREV samples. The other genera were grouped and named as “Others”.

**
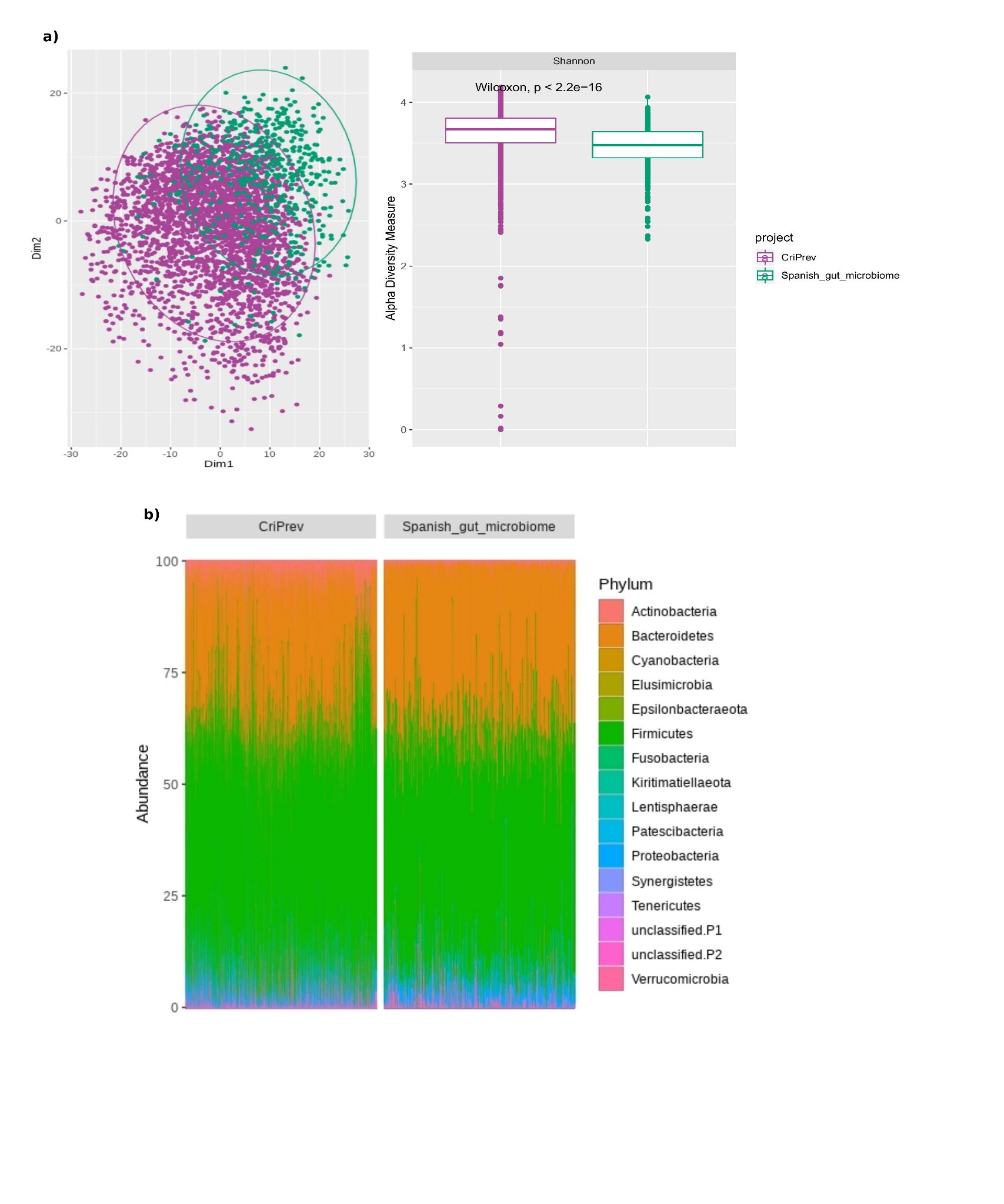
**

**Figure S2:** Comparison of FIT positive 16S samples from the present study and stool 16S samples from an independent study. A) Multidimensional scaling plot (MDS) representing the Aitchison distance and Shannon index according to the source project.

B) Barplot representing the present phyla. Each column represents a sample.


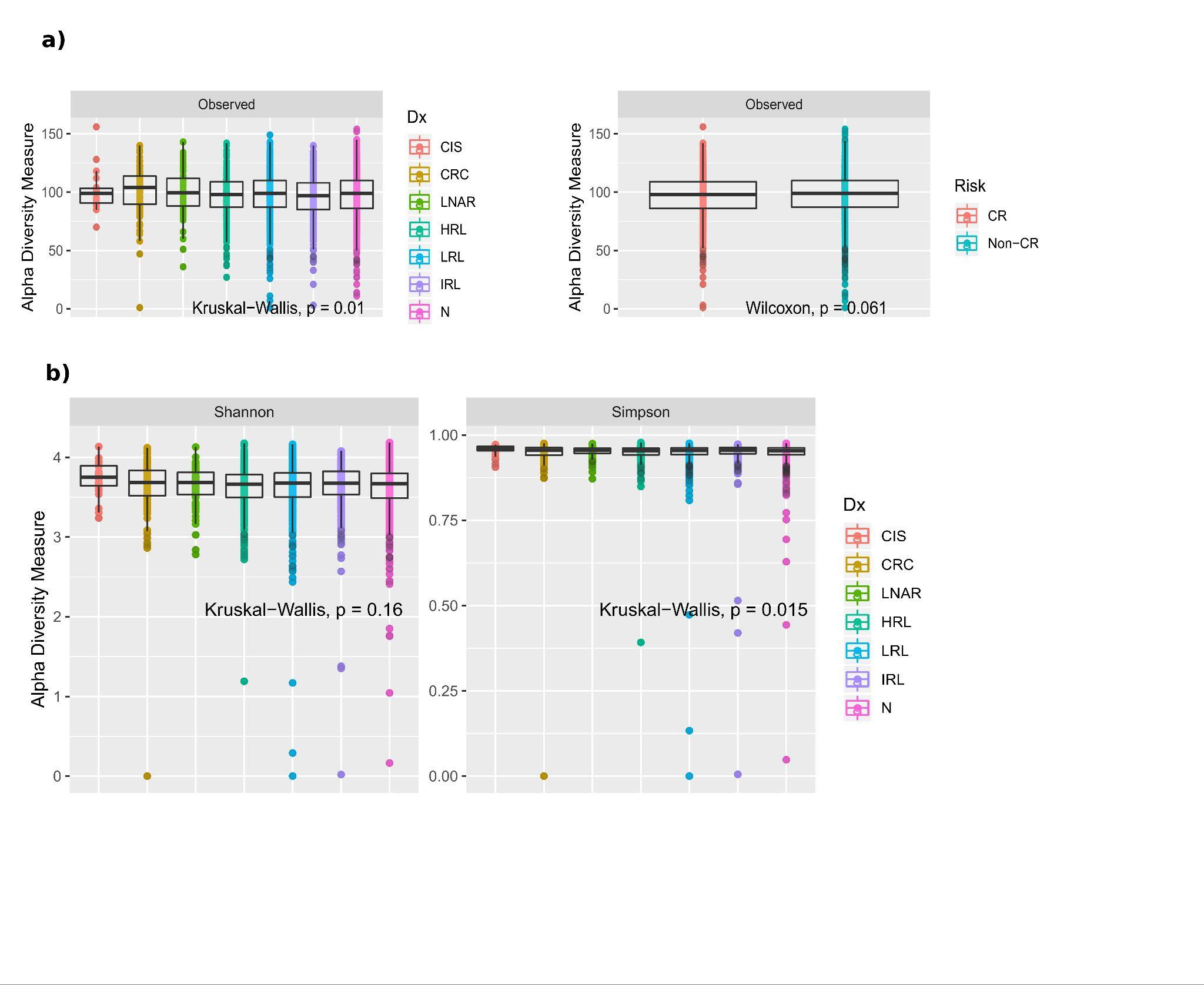


**Figure S3.** Alpha diversity characterization (n=2,889). The lines inside the boxplots represent the medians for each of the groups. Statistical test: Kruskall-Wallis or Wilcoxon test, with a significant result when p < 0.05. A) Observed index according to the diagnosis (carcinoma *in situ* (CIS), Colorectal cancer (CRC), lesion that is not associated to risk (LNAR), high risk lesion (HRL), low risk lesion (LRL), intermediate risk lesion (IRL) or Negative (N) samples) and Risk (clinically relevant (CR) vs non-clinically relevant (Non-CR) samples) variables. B) Shannon and Simpson indices according to the Diagnosis.


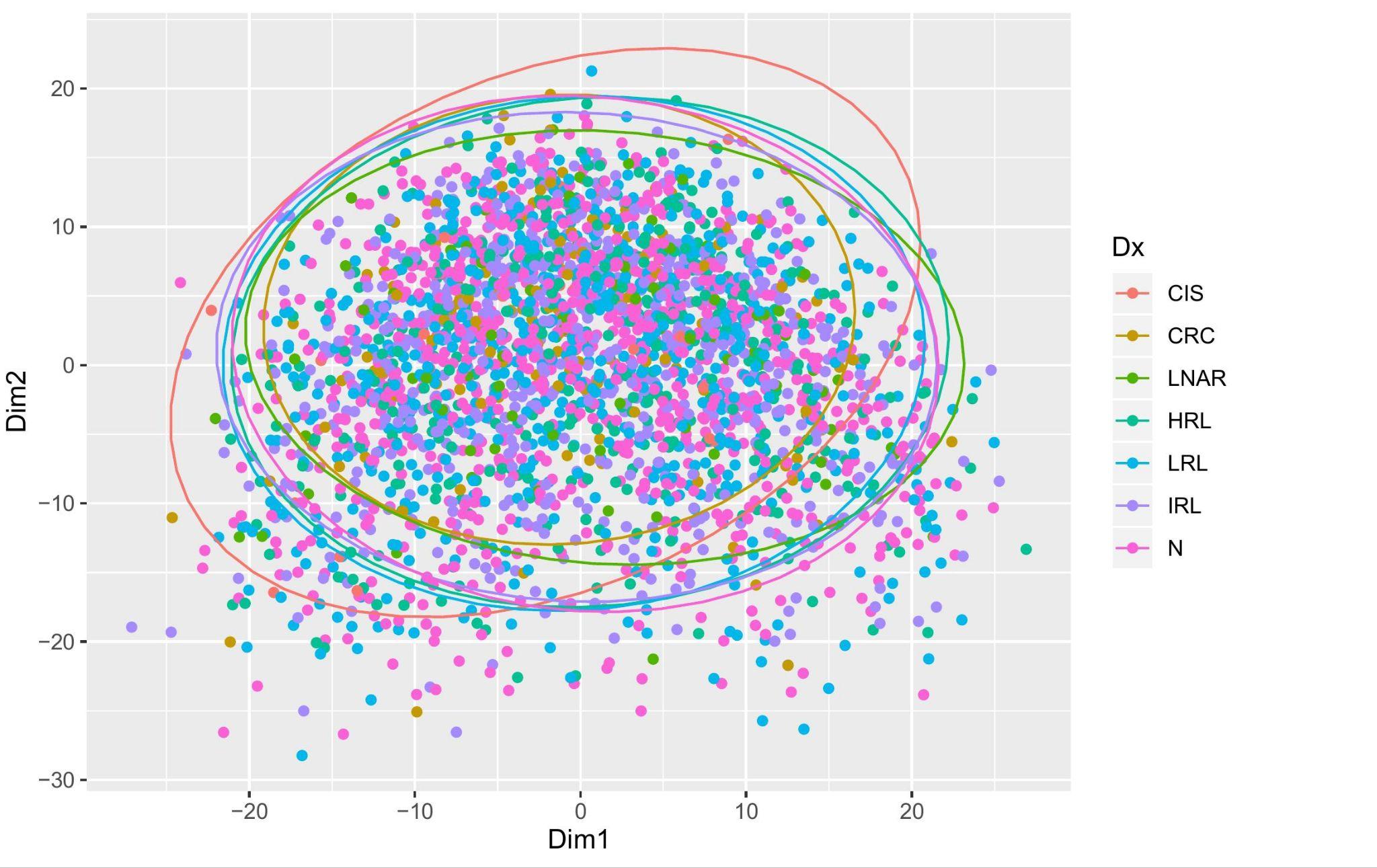


**Figure S4:** MDS plots using Aitchison distance (n=2,889). The samples are colored according to the diagnosis. 95% confidence ellipses are represented for each of the diagnosed groups.


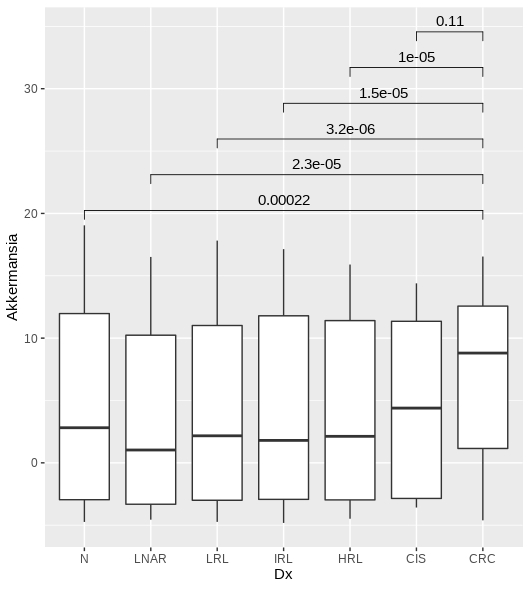


**Figure S5:** Box plot of the *Akkermansia* clr according to the different explored diagnosis (n=2,889). Negative (N), Lesion Not Associated to Risk (LNAR), Low Risk Lesion (LRL), Intermediate Risk Lesion (IRL), High Risk Lesion (HRL), Carcinoma in situ (CIS) and Colorectal Cancer (CRC). P-values between CRC and the other diagnoses are represented being all of them significant except CIS vs CRC.


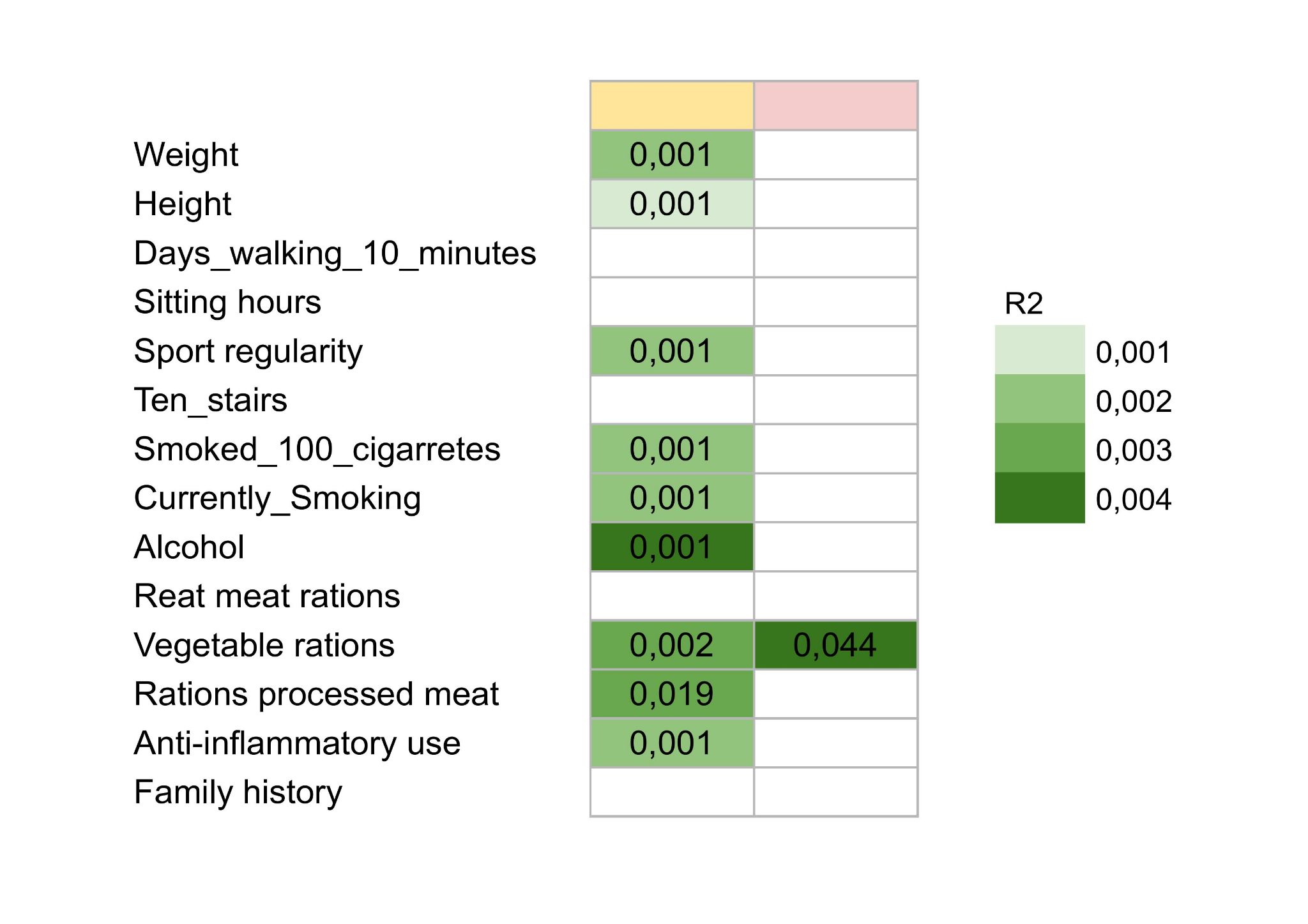


**Figure S6:** Summary of the results of the adonis test, evaluating the effect of lifestyle variables on the overall composition. Only significant (p-value < 0.05) results are colored, including the p-value in each of the cells. In the orange column there is the assessment of the individual effect of each variable while in the pink one its impact using as covariate the diagnosis. The explained variability (R2) was used for the color intensity of the cells.


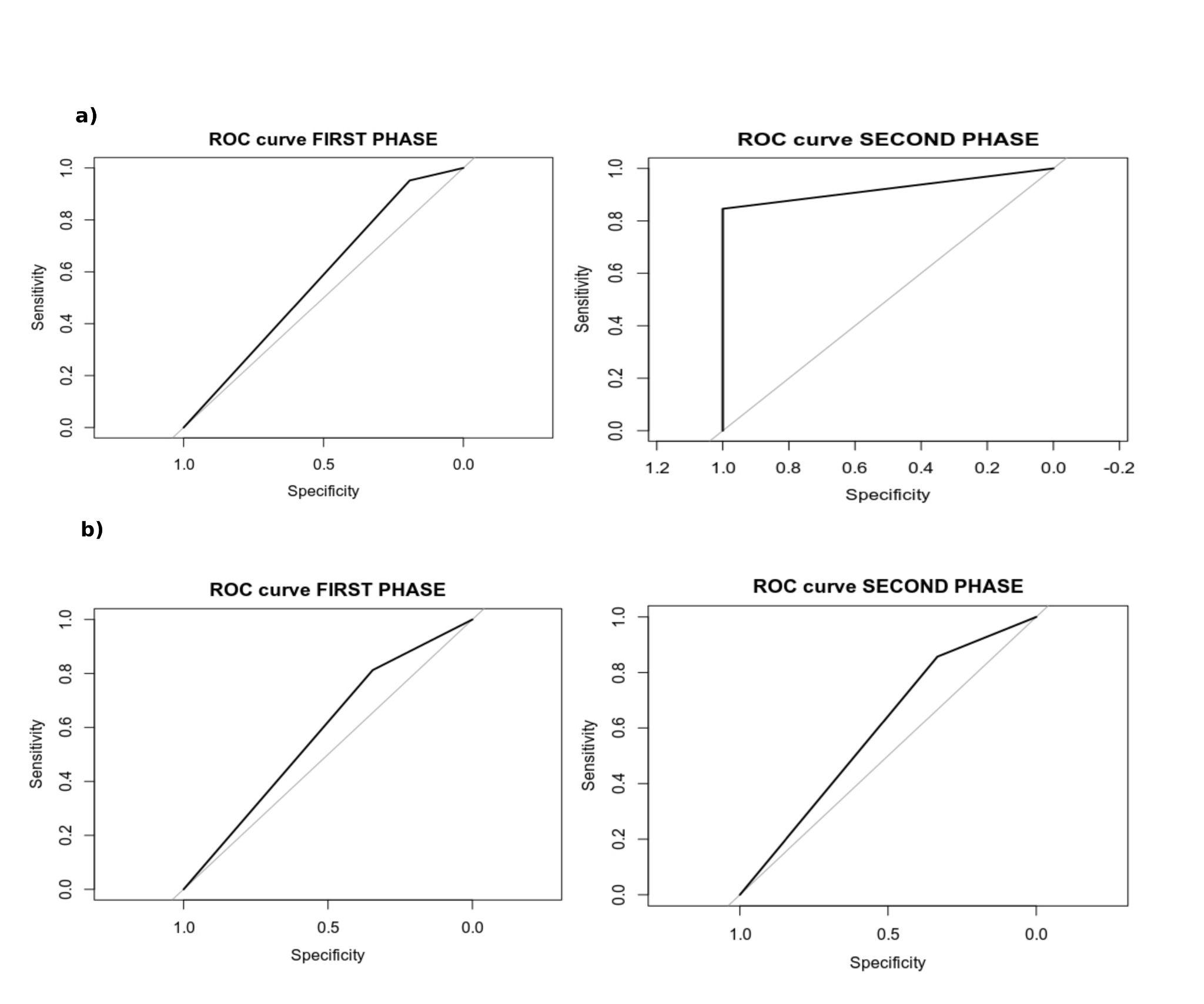


**Figure S7:** ROC curves for each of the phases in the different validations performed. FIRST phase: CRC vs Others, Second phase: Clinically relevant vs Non-Clinically relevant a) USA cohort. B) 100 extra samples from the CRC screening.


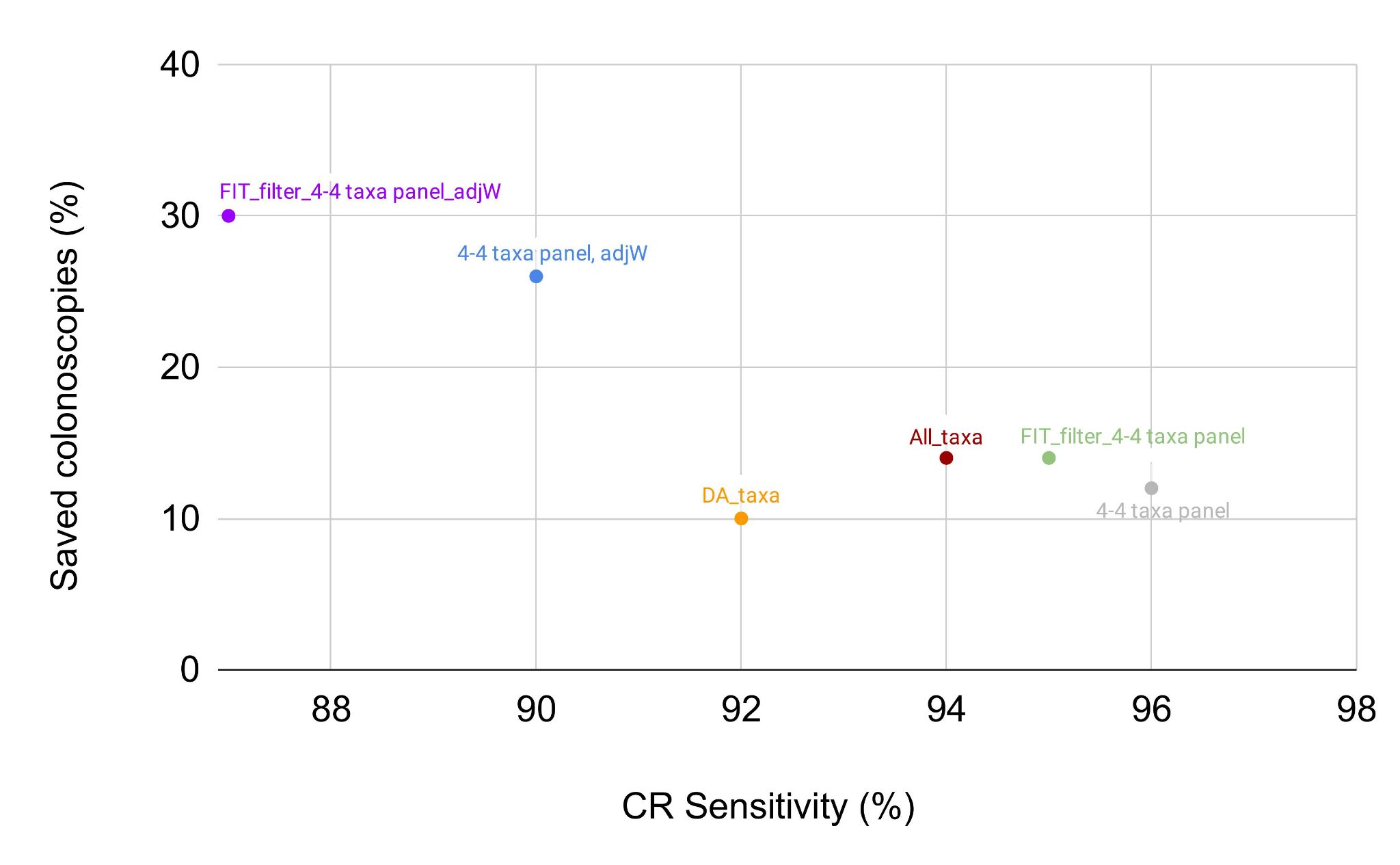


**Figure S8:** Percentage of saved colonoscopies and clinically relevant sensitivity according to the different specifications of the proposed classifier.

All_taxa: All the intersecting taxa between the CRIPREV and the validation datasets were used as features.

DA_taxa: All the intersecting differentially abundant taxa between the CRIPREV and the validation datasets were used as features.

4-4 taxa panel: 4 taxa panel for each of the phases.

4-4 taxa panel, adjW: 4 taxa panel for each of the phases, with less penalization of the CR samples in the second phase.

FIT_filter_4-4 taxa panel: Samples above 954 of the FIT value (μg hemoglobin/g feces) were directed to colonoscopy and the remaining samples were subjected to the classifier.

FIT_filter_4-4 taxa panel_adjW: Samples above 954 of the FIT value (μg hemoglobin/g feces) were directed to colonoscopy and the remaining samples were subjected to the classifier. Less penalization of the CR samples in the second phase.


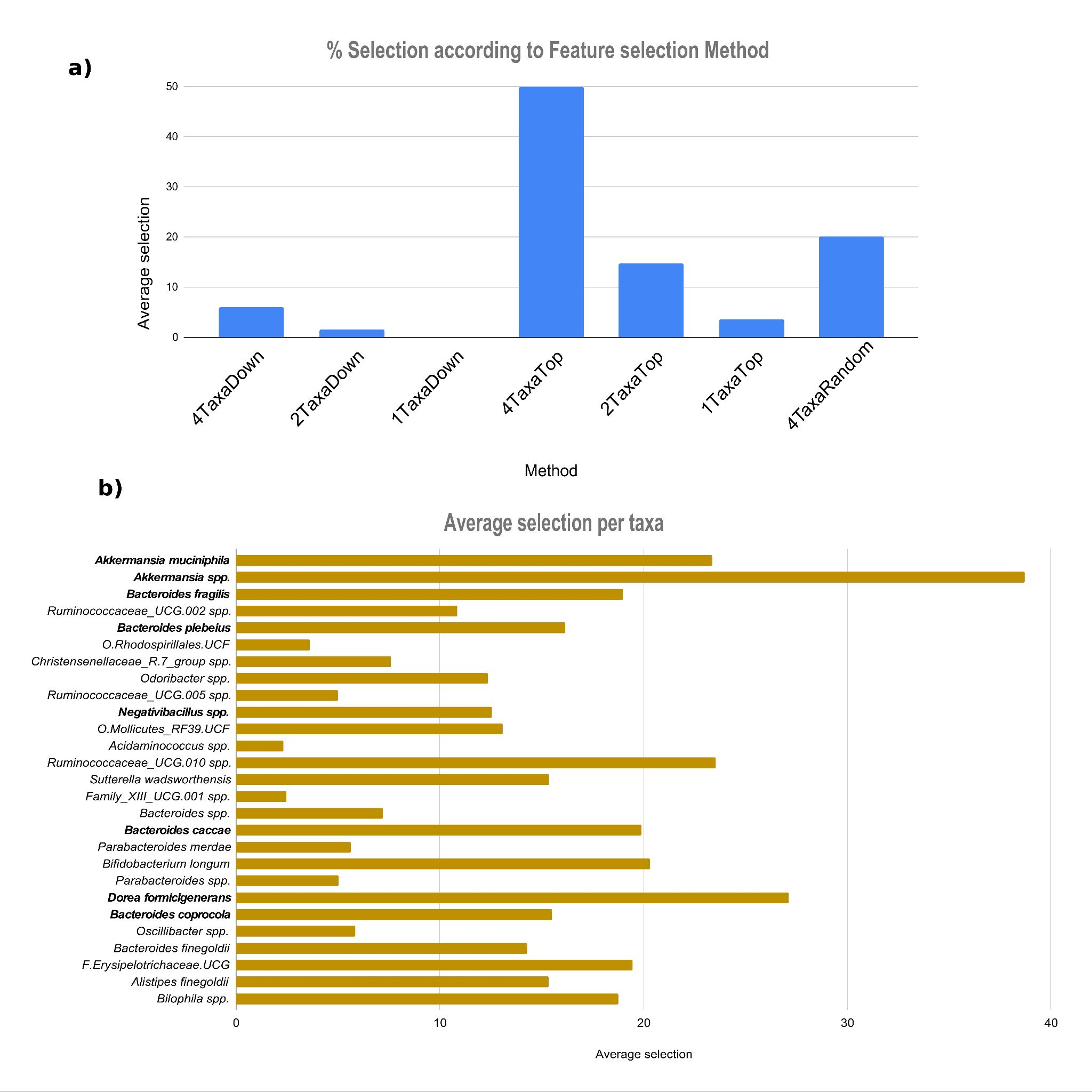


**Figure S9:** A) Potential selection (Number of models selected / Number of evaluated models, in %) of the different feature selection methods. B) Average potential selection of each of the 27 studied taxa (Number of selected models in which the taxa was included / Number of models in which the taxa was included as feature).

**SUPPLEMENTARY TABLES**

**Table S1.** Criteria and distribution of the colonoscopy-based diagnosis types considered in this project. Columns indicate, in this order, the diagnosis group, the criteria for classification in the group, the number of samples of this study in the given group, and the clinical relevance.

| **Diagnosis group** | **Criteria** | **Samples (n)** | **Clinical relevance** |
| --- | --- | --- | --- |
| Negative (N) | Absence of adenomas or polyps | 925 | Non-CR |
| Lesion Not Associated to Risk (LNAR) | < 20 hyperplastic polyps < 10 mm limited to rectal or sigmoid colon | 90 | Non-CR |
| Low Risk Lesion (LRL) | 1-2 tubular adenomas < 10 mm with low-grade dysplasia or 1-2 serrated polyps < 10 mm without dysplasia | 681 | Non-CR |
| Intermediate Risk Lesion (IRL) | 3-4 tubular adenomas <10 mm with low-grade dysplasia or  1-4 tubular adenomas 10-19 mm with low-grade dysplasia or  1-4 adenomas < 20 mm with villous component and/or high-grade dysplasia (intraepithelial carcinoma) and/or intramucosal carcinoma, or  3-4 serrated polyps <10 mm without dysplasia, or  1-4 serrated polyps 10-19 mm without dysplasia, or  1-4 serrated polyps < 20 mm with dysplasia. | 638 | CR |
| High Risk Lesion (HRL) | >= 5 Adenomas/ Serrated polyps, or  >= 1 Adenomas/ Serrated polyps >=20 mm | 397 | CR |
| Carcinoma *in situ* (CIS) | Non invasive, intramucosal carcinoma. Stage 0. | 24 | CR |
| Colorectal cancer (CRC) | Invasive colorectal adenocarcinoma. From Stage I to IV. | 134 | CR |

**Table S2.** Characteristics of the included individuals: Sex, median and range age and samples deemed of clinical relevance after colonoscopy. *Samples with ‘NA’ value for this parameter are excluded from the calculation.

| **Sex** | **Individuals** | | **Median and Age range*** | **Clinically relevant lesions** | |
| --- | --- | --- | --- | --- | --- |
|  | **N** | **%** |  | **N** | **%** |
| All | 2,889 | 100 | 60 [49,71] | 1193 | 41,29 |
| Males | 1548 | 53.58 | 60 [49,71] | 742 | 47.93 |
| Females | 1341 | 46.42 | 61 [49,70] | 451 | 33,63 |

**Table S3.** Table summarizing differential abundance analysis results considering all the diagnoses following the path from healthy colon to colorectal cancer. Used linear model: Tax_element ~ Diagnosis + HOSPITAL + SEX + AGE + N_POLYPS + FIT_VALUE + (1|RUN).Samples with missing metadata were not considered in this analysis ( n=2,565).

|  | **Phylum** | **Class** | **Order** | **Family** | **Genus** | **Species** |
| --- | --- | --- | --- | --- | --- | --- |
| **Diagnosis** | 3 | 4 | 6 | 10 | 18 | 34 |
| **Hospital** | 6 | 10 | 14 | 27 | 73 | 112 |
| **Sex** | 4 | 7 | 13 | 30 | 96 | 132 |
| **Age** | 2 | 3 | 7 | 15 | 42 | 78 |
| **N_polyps** | 1 | 2 | 2 | 4 | 15 | 33 |
| **FIT_value** | 1 | 1 | 1 | 4 | 10 | 14 |

**Table S4.** Performance of the two-phase machine learning predictor. The reported values are mean values obtained from the 100 random splits. Including 41 and 34 taxa for both phase 1 and phase 2, respectivetly, plus sex, age and fecal hemoglobin concentration.Samples with missing metadata were discarded from this analysis (n=2,817). A) Average of Area Under the Curve (AUC), Recall and Specificity for each of the phases. B) Average sensitivity for clinically relevant samples and for each of the diagnoses included in this particular group.

|  | **AUC** | **Recall** | **Specificity** |
| --- | --- | --- | --- |
|  |  |  |  |
| **FIRST PHASE** | 0.618836 | 0.8048148 | 0.4328572 |
| **SECOND PHASE** | 0.5488568 | 0.7279377 | 0.369776 |

B)

|  | **Average sensitivity (%)** |
| --- | --- |
| **CR** | 95.91 |
| **IRL** | 95.57 |
| **HRL** | 95.84 |
| **CIS** | 97.42 |
| **CRC** | 97.59 |

**Table S5.**Performance of the two-phase machine learning predictor on independent datasets. The reported values are obtained by training on all the CriPrev samples (samples with missing metadata were discarded for training the model, n=2,817) and testing on the independent sets. Area Under the Curve (AUC), Recall and Specificity for each of the phases and sensitivity for CRC and CR lesions at the end of the two-phase classification were reported. A) USA cohort. Including a panel of 3 and 4 taxa for phase 1 and 2, respectively, plus sex, age and fecal hemoglobin concentration. B) 100 extra samples from the Catalan screening.

A)

|  | **AUC** | **Recall** | **Specificity** | **TWO-PHASE CLASSIFIER** | | |
| --- | --- | --- | --- | --- | --- | --- |
|  |  |  |  | **Sensitivity for CRC**  **(%)** | **Sensitivity for CR lesions**  **(%)** | **Saved colonoscopies (%)** |
| **FIRST PHASE** | 0.5721 | 0.9518 | 0.1923 |  |  |  |
| **SECOND PHASE** | 0.9231 | 0.8462 | 1 | 100 | 98.46 | 20 |

B)

|  | **AUC** | **Recall** | **Specificity** | **TWO-PHASE CLASSIFIER** | | |
| --- | --- | --- | --- | --- | --- | --- |
|  |  |  |  | **Sensitivity for CRC**  **(%)** | **Sensitivity for CR lesions**  **(%)** | **Saved colonoscopies (%)** |
| **FIRST PHASE** | 0.5789 | 0.8125 | 0.3452 |  |  |  |
| **SECOND PHASE** | 0.5952 | 0.8571 | 0.3333 | 100 | 96 | 12 |

**Table S6.** Comparison of our algorithm (considering different optimizations, shadowed cells) with two alternative solutions and the current FIT strategy.

| **Strategy** | **Sample** | **CRC sensitivity (%)** | **CR sensitivity (%)** | **False positive rate (%)** |
| --- | --- | --- | --- | --- |
| 4-4 taxa panel classifier | Self-sample.  Same sample than FIT test | 100 | 96 | 53 |
| 4-4 taxa panel, adjusted weights | Self-sample.  Same sample than FIT test | 100 | 90 | 39 |
| FIT_filter_4-4 taxa panel, adjusted weights | Self-sample.  Same sample than FIT test | 100 | 87 | 35 |
| GoodGut | Need fresh sample. Only for this test | 94.70 | 82.3 | 48.7 |
| ColoGuard | Full deposition.  Home sample. | 92.3 | 42.4 | 13 |
| FIT | Self-sample. Same sample than FIT test | 73.8 | 23.8 | 65 |

**LEGENDS FOR SUPPLEMENTARY DATA**

**Data S1.** Strengthening The Organization and Reporting of Microbiome Studies (STORMS) checklist

**Data S2.** Metadata will be available prior to publication.

**Data S3.** Table of the taxa at species level that we found as differentially abundant according to each of the fixed effects included in the linear model. Only significant p-values are reported. Samples with missing metadata were not considered in this analysis. (n=2,565).

**Data S4.** Table of the taxa at species level that we found as differentially abundant according to each of the fixed effects included in the linear model when comparing CRC vs Non-CRC. Only significant p-values are reported. Samples with missing metadata were not considered in this analysis. (n=2,565).

**Data S5.** Table of the taxa at species level that we found as differentially abundant according to each of the fixed effects included in the linear model when comparing Clinically relevant (CR) vs Non-Clinically relevant (Non-CR) samples. Only significant p-values are reported. Samples with missing metadata were not considered in this analysis. (n=2,565).

**Data S6.** Table of species found as differentially abundant according to the number of polyps, and the significance values (P-value < 0.05). Samples with missing metadata were not considered in this analysis. (n=2,565).

**Data S7.** List of differentially abundant OG according to the diagnosis and the significance values (P-value < 0.05).

**Data S8**. Summary of the significant results obtained when applying multiple comparisons between diagnoses. Significant p values are reported (Tukey test, p.adjusted < 0.05). The p-value has the sign of the corresponding effect size, indicating the direction of the difference.

**Data S9.** Statistics of the last 4 available rounds of results from the Catalan CRC screening in Barcelona.
